# Haplotype Block Analysis Reveals Candidate Genes and QTLs for Meat Quality and Disease Resistance in Chinese Jiangquhai Pig Breed

**DOI:** 10.3389/fgene.2020.00752

**Published:** 2020-09-04

**Authors:** Favour Oluwapelumi Oyelami, Qingbo Zhao, Zhong Xu, Zhe Zhang, Hao Sun, Zhenyang Zhang, Peipei Ma, Qishan Wang, Yuchun Pan

**Affiliations:** ^1^Department of Animal Science, School of Agriculture and Biology, Shanghai Jiao Tong University, Shanghai, China; ^2^Department of Animal Breeding and Reproduction, College of Animal Science, Zhejiang University, Hangzhou, China

**Keywords:** linkage disequilibrium, haplotypes, conservation, complex traits, Chinese pigs

## Abstract

The Jiangquhai (JQ) pig breed is one of the most widely recognized pig populations in China due to its unique and dominant characteristics. In this study, we examined the extent of Linkage disequilibrium (LD) and haplotype block structure of the JQ pig breed, and scanned the blocks for possible genes underlying important QTLs that could either be responsible for some adaptive features in these pigs or might have undergone some selection pressure. We compared some of our results with other Chinese and Western pig breeds. The results show that the JQ breed had the highest total block length (349.73 Mb ≈ 15% of its genome), and the coverage rate of blocks in most of its chromosomes was larger than those of other breeds except for *Sus scrofa* chromosome 4 (SSC4), SSC6, SSC7, SSC8, SSC10, SSC12, SSC13, SSC14, SSC17, SSC18, and SSCX. Moreover, the JQ breed had more SNPs that were clustered into haplotype blocks than the other breeds examined in this study. Our shared and unique haplotype block analysis revealed that the Hongdenglong (HD) breed had the lowest percentage of shared haplotype blocks while the Shanzhu (SZ) breed had the highest. We found that the JQ breed had an average *r*^2^ > 0.2 at SNPs distances 10–20 kb and concluded that about 120,000–240,000 SNPs would be needed for a successful GWAS in the breed. Finally, we detected a total of 88 genes harbored by selected haplotype blocks in the JQ breed, of which only 4 were significantly enriched (*p-value* ≤ 0.05). These genes were significantly enriched in 2 GO terms (*p-value* < 0.01), and 2 KEGG pathways (*p-value* < 0.02). Most of these enriched genes were related to health. Also, most of the overlapping QTLs detected in the haplotype blocks were related to meat and carcass quality, as well as health, with a few of them relating to reproduction and production. These results provide insights into the genetic architecture of some adaptive and meat quality traits observed in the JQ pig breed and also revealed the pattern of LD in the genome of the pig. Our result provides significant guidance for improving the statistical power of GWAS and optimizing the conservation strategy for this JQ pig breed.

## Introduction

The pig population in China (435 million) accounts for 45% of the total population of pigs in the world (FAOSTAT, 2017) and the Jiangquhai (JQ) pig breed is one of the most widely recognized pig populations in the country due to its unique and dominant characters. This pig breed is found in Jiangsu Province, in the eastern part of China where the giant Taihu lake is located. The JQ breed is known for its high performing economic traits like reproduction, adaptability, disease resistance, and the quality of its meat (China National Commission of Animal Genetic Resources, 2011).

The JQ pig breed has existed since the early 19th century and has many characteristics such as strong fat deposition and excellent tasting, high-quality meat. It is a well known local pig breed used in producing ham in China (China National Commission of Animal Genetic Resources, 2011), where there are three popular types of ham: Yun ham, Jinhua ham, and Rugao ham. While Yun hams are produced from three Yunnan province pig breeds, Jinhua ham is produced from Jinhua pig, and Rugao ham is produced from Jiangquhai (JQ) pig (Miao et al., 2009; Toldrá et al., 2014). Apart from JQ pigs, other pig populations such as Huaibei (HB), Hongdenglong (HD), Shanzhu (SZ), Dongchuan, Erhualian, Fengjing, Huai, Mi, and Shawutou are also distributed throughout Jiangsu province.

Recent studies had revealed high genetic diversity within the JQ pig breed (Hua et al., 2014; Xiao et al., 2017b; Xu et al., 2019). Genomic analysis also revealed that this breed might have undergone selection in the past, which could account for some adaptive traits in the breed (Meng et al., 2018; Xu et al., 2019). However, there is still a dearth of information on the genetic architecture of some economically important traits in this pig breed. Moreover, the adaptation of this breed to its environment is strongly supported by empirical evidence indicating that the genetic basis of its population differentiation is non-additive for fitness trait and that its adaptive gene complexes would be different from those of other breeds (Crnokrak and Roff, 1995). Therefore, it is imperative to understand the non-random genetic relationship between loci within the JQ pig population. This relationship is usually reflected by the pattern and extent of linkage disequilibrium (LD) that are inferred from the haplotypes in the genome.

Advancement in high-throughput genotyping technologies enables the use of large numbers of single nucleotide polymorphism (SNPs) in detecting haplotypes, which are products of introgression or selection during the domestication process of pigs (Amaral et al., 2008). These haplotypes can be inherited from one generation to the other as single units called haplotype blocks (Gabriel et al., 2002). Haplotype blocks are sections of the chromosome with high LD, low haplotype diversity, and low recombination rate (Luikart et al., 2003; Phillips et al., 2003). Many haplotype blocks may arise as a result of several factors such as chromosomal recombination, selection, population bottlenecks, population admixture, and mutations (Phillips et al., 2003; Guryev et al., 2006). Previous studies have reported a low level of admixture in the JQ pig breed, however, the degree of admixture of this breed by possible sources of admixtures is unknown. Therefore, the identification of the percentage of foreign haplotypes in the JQ breed could serve as a useful framework of future breeding actions and decisions when setting up a conservation program for the breed. Moreover, since the evolutionary history of a breed can be inferred from the pattern of LD in the genome (Hayes et al., 2003), the characterization of the patterns of LD across the genome of JQ pigs could potentially improve our understanding of the biological pathway of recombination in the breed, and also help to detect some selection footprints in the genome. Furthermore, characterizing the LD structure in the genome is particularly important for the interpretation and application of results of genome-wide association studies (GWAS) (Meuwissen and Goddard, 2000).

Over the years, haplotypes have proven to be more powerful in association studies than single-marker methods (Lin et al., 2009). Thus, they have a point of reference in GWAS, especially in the case of ungenotyped SNPs. Haplotype blocks can be used to identify significant variants in GWAS and also for predicting the genomic breeding values (GEBV) of animals in genomic selection (GS) programs (Meuwissen et al., 2001; Calus et al., 2008; Cuyabano et al., 2015; Chen et al., 2018). Therefore, the characterization of LD patterns in the genome of JQ pigs has a potential application in future studies of complex traits and the development of genomic tools for the breed (Corbin et al., 2010).

Since the extent of LD and haplotype blocks are of critical importance for genomic selection, marker-assisted selection, and conservation of animal genetic resources, the importance of constructing the haplotype blocks in the JQ pig breed and identifying the genes involved in them, especially those associated with economically important traits, cannot be overemphasized. Such information would help in understanding the genetic basis of breed distinction and adaptation and guide against incorporating haplotype blocks with deleterious gene effects into selection programs (Salem et al., 2018). To our knowledge, no haplotype block study has been conducted on this pig breed despite its unique characteristics and there is still a knowledge gap on the genetic basis of its phenotypic distinction. To this end, this research was conducted to (1) analyze the haplotype block structure of the JQ pig breed and compare it with seven other pig breeds (five Chinese and two western breeds), (2) examine the pattern of linkage disequilibrium (LD) in the JQ breed, and (3) scan the blocks for possible genes underlying important QTLs that span across the blocks. Our result provides a theoretical basis for designing breeding programs aimed at conserving economically important traits in the JQ breed and potential genetic improvement programs for this breed in the future.

## Materials and Methods

### Animal Samples, Genotyping and Quality Control

A total of 192 pigs were used in this study. Of the total pig population, thirty-eight (38) were Jiangquhai (JQ) pigs from the pig conservation farm in Jiangsu province. Other pig breeds used as a reference population were; Huaibei (HB, *n* = 34), Shanzhu (SZ, *n* = 20), and Hongdenglong (HD, *n* = 30) breeds, also from Jiangsu Province; Middle Meishan (MMS, *n* = 20) and PudongWhite (PD, *n* = 20) pigs from Shanghai province; and, Duroc (D, *n* = 10) and Yorkshire (Y, *n* = 20), which are western pig breeds. The Jiangsu pig samples from the conservation pig farms have been described in previous studies (Xiao et al., 2017b; Zhang et al., 2018). In these previous studies, the individuals were genotyped using the genotyping by genome reducing and sequencing (GGRS) protocol^1^ (Chen et al., 2013). Briefly, genomic DNA samples were extracted from ear tissue, using a Lifefeng blood and tissue extraction kit [Lifefeng Biotech (Shanghai) Co., Ltd., China], digested with a restriction enzyme (*Ava*II), and then ligated with a unique adapter barcode after which the samples were pooled and enriched through PCR to construct a sequencing library. Finally, the DNA sequence libraries (fragments lengths of 300–400 bp, including the adapter barcode sequence) were sequenced using an Illumina HiSeq2500 (100 paired-end) sequencing platform according to the manufacturer’s protocol.

Quality control of sequences was performed using NGS QC Toolkit v2.3 and the parameters were set according to a report from Chen et al. (2013). The sequencing reads were aligned to the pig reference genome (Sscrofa11.1) using BWA (Li and Durbin, 2009). The BAM files from the alignments were used to call and genotype SNPs using SAMtools (Li et al., 2009). These variants were then filtered and SNPs with a quality score greater than or equal to 20 (i.e., more than 99% accuracy), average sequencing depth > 5x, and minor allele frequency (MAF) greater than or equal to 0.03 were retained for imputation (Chen et al., 2013; Wang et al., 2015). To ensure the precision of imputation and density of SNPs, only those genotyped in >30% of samples were retained (Wang et al., 2015). BEAGLE v4.1 was used to impute the missing genotypes in this study with default parameters (Browning and Browning, 2016). A total of 486,018 SNPs, which passed the filtering threshold, were later separated into different populations and filtered for MAF ≥ 0.05. After discarding SNPs on the Y Chromosome, a total of 270,935, 223,897, 317,597, 210,277, 204,790, 237,962, 173,678, and 221,957 SNPs, with MAF ≥ 0.05, were retained in JQ, HB, SZ, HD, MMS, PD, D and Y breed, respectively. The alignment and variant calling statistics are presented in Supplementary Tables S1, S2.

### Genetic Relationships and Population Structure

To estimate the genetic distances within breeds, the average proportion of alleles shared, Dst, was calculated using PLINK v1.9 (Chang et al., 2015). The definition of Dst is as follows (Chang et al., 2015):

$$D\,s\,t=\frac{I\,B\,S_{2}+0.5^{*}\,I\,B\,S_{1}}{N}$$

IBS_1_ and IBS_2_ are the numbers of loci that share 1 or 2 alleles identical by state (IBS), respectively, and N is the number of loci tested. The genetic distance (D) between all pairwise combinations of individuals was calculated as follows: 1-Dst. Pairwise genetic differentiation (fixation index, *F*_*ST*_) (Weir and Cockerham, 1984) between all pairs of pig breeds were calculated using the R package “diversity” (Keenan et al., 2013). Based on the matrix of pairwise *F*_*ST*_ values, a Neighbor-Net tree was constructed using SplitsTree 4.14.5 software (Huson and Bryant, 2006).

To illustrate the population structure and infer genetic admixture between populations, a total of 91,092 SNPs, which discarded SNPs that were with extreme deviations from Hardy-Weinberg equilibrium (*p*-value ≤ 1 × 10^–6^), MAF < 0.05, and LD (linkage disequilibrium) greater than 0.5 across populations (command: PLINK indep-pairwise 50 5 0.5), were used for population structure analysis using ADMIXTURE v1.3 software (Alexander et al., 2009). The number of ancestral clusters (K) was set from 2 to 9, and a five-fold cross-validation was run to determine the *K* value with the lowest cross-validation error. The result was displayed using the web-based software, Clumpak^2^ (Kopelman et al., 2015).

### Effective Population Size

The historical effective population size (*N*_*e*_) of each breed was estimated using the SNP data from the admixture analysis. *N*_*e*_ was estimated using the software SNeP (Barbato et al., 2015). SNeP estimates *N*_*e*_ at different *t* generations based on the LD between SNPs, where *t* = [2*f*(*c*_*t*_)]^–1^, and *c*_*t*_ is the recombination rate for specific physical distance between markers, measured in Morgan (Hayes et al., 2003) (assuming 100 Mb = 1Morgan). The following options were also used in SNeP: (1) sample size correction; (2) correction to account for the occurrence of mutation; (3) Sved and Feldman’s recombination rate modifier (Sved and Feldman, 1973).

### Linkage Disequilibrium

Linkage disequilibrium, *r*^2^ value was used as a measure of LD between each locus because of its preference in association studies (Wall and Pritchard, 2003; Bohmanova et al., 2010). We estimated pairwise LD (*r*^2^) for all retained SNPs within each breed using the command line “–ld- window-r2 0” in PLINK v1.9 (Chang et al., 2015). This procedure used a default maximum window size of 1 Mb between the estimated pair of SNPs on a chromosome. The extent and decay of LD in each breed were also predicted using the following equation (Sved, 1971; Heifetz et al., 2005; Amaral et al., 2008; Ai et al., 2013):

$$L\,D_{i\,j\,k}=\frac{1}{1+4\,\beta_{j\,k}\,d_{i\,j\,k}}+e_{i\,j\,k}$$

Where LD*_*ijk*_* is the observed LD for marker pair *i* of breed *j* in genomic region *k*, d*_*ijk*_* is the distance in base pairs for marker pair *i* of breed *j* in genomic region *k*, β*_*jk*_* is the coefficient that describes the decline of LD with distance for breed *j* in genomic region k and e*_*ijk*_* is a random residual. The LD*_*ijk*_*, β*_*jk*_*, and e*_*ijk*_* for each genomic region within each breed were estimated using the Beta.nonlinear fit function in *R*^3^ (Amaral et al., 2008). They were fitted for the following genomic distances; 0, 4, 8, 12, 20, 30, 40, 60, 80, 100, 120, 160, 200, 250, 300, 360, 460, 620, 800, and 1000 kb. The decay of LD was plotted for both, autosomes and SSCX of each breed. To further assess the extent of LD across breeds, the LD (*r*^2^) between all autosomal SNPs was, however, divided into the following bin distances: 0–10, 10–20, 20–40, 40–60, 60–100, 100–200, 200–500, and 500–1000 kb.

### Haplotype Block Construction and Haplotype Diversity

A Hidden Markov Model implemented in the program BEAGLE v4.1 software (Browning and Browning, 2016) was used to reconstruct the haplotype phase. Hereafter, haplotype blocks were estimated separately in each breed using PLINK v1.9 (Chang et al., 2015) following the default procedure in HAPLOVIEW (v4.1) (Barrett et al., 2005). The method followed for block definition was previously described by Gabriel et al. (2002). Furthermore, to investigate the pattern of LD within blocks, a haploview plot was constructed for some haplotype blocks, based on LD (*r*^2^) value between SNP pairs, using the HAPLOVIEW software (Barrett et al., 2005).

As a measure of genetic diversity, we estimated haplotype diversity across breeds. First, we calculated the haplotype frequency for each breed using PLINK v1.07 (Purcell et al., 2007) (because PLINK v1.9 does not currently support the –hap flag which is needed to calculate the haplotype frequency). Afterward, the haplotype diversity across breeds was estimated. Haplotype diversity is defined as $1-\sum f_{i}^{2}$ where *f*_*i*_ is the frequency of the ith haplotype. To gain insight into the haplotype diversity within the block region (with the maximum number of SNPs in JQ breed), we applied the “four-gamete rule block definition algorithm” implemented in haploview software. This algorithm computes the observed frequency of the four possible two-marker haplotypes for each pair of SNPs and defines a block when the frequency is 0 (i.e., no recombination event has occurred) (Wang et al., 2002). A frequency of at least 0.01 between computed four marker-haplotypes indicates that a recombination event between the two markers likely occurred.

The shared and unique haplotype block regions between breeds were also detected. Shared haplotype blocks were defined as the overlapping block regions shared by two populations or more, while the unique haplotype blocks were the block regions specific to each population. Both the shared and unique haplotype block regions were detected and visualized using the R Bioconductor package “GenomicRanges” (Lawrence et al., 2013) and ‘ggbio’ (Yin et al., 2012), respectively.

### QTLs and Functional Gene Set Enrichment Analysis

To detect the possible genes and important QTLs that span the haplotype blocks, we hypothesized that important traits under selection for adaptation of the JQ pig breed could be harbored in haplotype block regions with the highest block length. We also theorized that haplotype blocks with the highest number of SNPs could reveal some important genetic variations in the JQ breed. Therefore, we chose the first ten haplotype block regions within each respective criterion for functional annotation. In total, 20 block regions were annotated for possible QTLs and genes.

The QTL regions spanned by the haplotype block of the JQ breed were detected by mapping selected haplotype block regions onto QTL sections using data from the Pig QTL database^4^. To ensure efficient processing and control the volume of QTLs detected, we filtered out QTL regions to lengths ≤ 10 Mb, afterward, a Perl homemade script was used to detect haplotype block regions with more than 50% overlap with the filtered QTL regions. The QTLs that fall within the selected haplotype block regions or the haplotype block regions that fall within the QTLs are defined as overlap.

Furthermore, we performed a gene set enrichment analysis (GSEA) to further elucidate the biological function of the selected haplotype block regions above. We mapped the selected haplotype block regions and genes using gene annotation data for pigs from the Ensembl gene database 98^5^. Thereafter, the detected genes were functionally annotated by performing the Kyoto Encyclopedia of Genes and Genomes (KEGG) pathway (Kanehisa et al., 2012) and Gene Ontology (GO) (Ashburner et al., 2000) enrichment analysis using Database for Annotation, Visualization and Integrated Discovery (DAVID v6.8)^6^ (Huang et al., 2009). We defined a significant threshold *p-*value to be 0.05 (based on EASE score: a modified Fisher’s exact test), and then selected the most significantly enriched genes with FDR (False Discovery Rate) < 15%. We also established the relationship between the likely candidate genes and QTLs detected in the haplotype blocks. This result potentially reveals the genes and characters that might have either undergone artificial or natural selection pressure in the JQ breed or the genes involved in complex traits of the breed.

## Results

### Genetic Relationship and Population Structure

**FIGURE 1:**
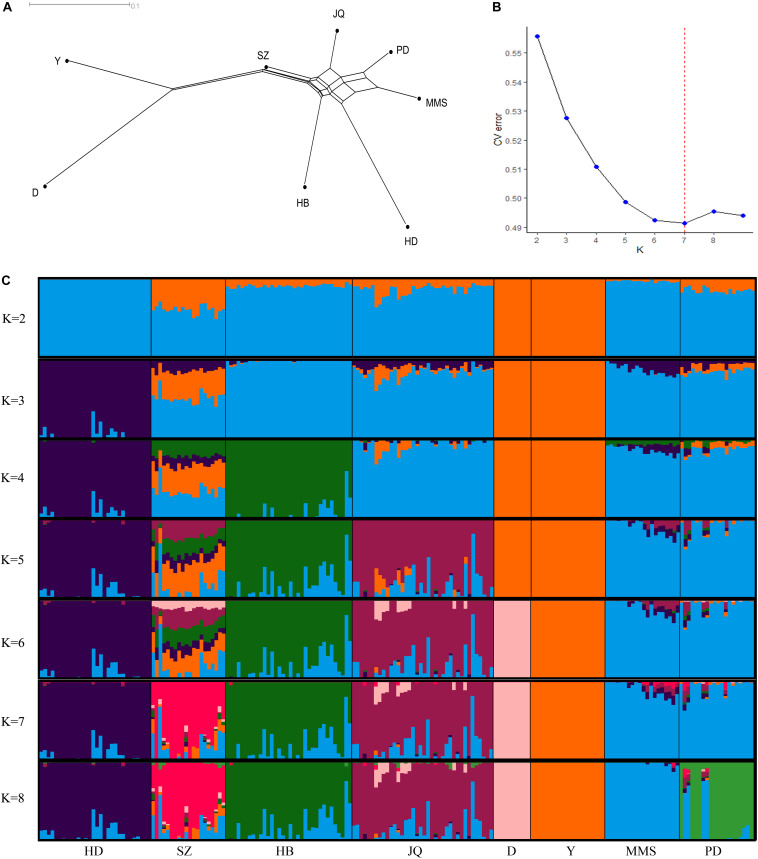
Population structure of the studied pigs. **(A)** Neighbor-Net tree showing genetic differentiation (Fst) between breeds. **(B)** Cross-validation errors of the admixture analysis at different *K* values (lowest *k*-value = 7). **(C)** Population structure of the 8 breeds analyzed based on Admixture analysis. Hongdenglong (HD), Shanzhu (SZ), Huaibei (HB), Jiangquhai (JQ), Duroc (D), Yorkshire (Y), MiddleMeishan (MMS), and PudongWhite (PD).

The average genetic distances (Dst) within the 8 populations were 0.210 (JQ), 0.181 (HD), 0.198 (HB), 0.255 (SZ), 0.164 (MMS), 0.203 (PD), 0.133 (D), and 0.167 (Y). The highest genetic differentiation (0.441) between breeds was found between MMS and D breed, while the lowest was found between MMS and PD breed (0.093) (Supplementary Table S3). A Neighbor-Net tree constructed based on this pairwise *F*st value between breeds is presented in Figure 1A. Our admixture analysis revealed some level of introgression between the Chinese breeds in this study (Figure 1C). We observed the lowest cross-validation error when *K* = 7 (Figure 1B), before PD separated from MMS into a different cluster. Suggesting a continuous gene flow between PD and the MMS breed (Xiao et al., 2017a). Consistent with previous findings and the genetic origins of worldwide pig breeds (Fan et al., 2002; Ai et al., 2013; Zhang et al., 2018), *K* = 2 shows the ancient divergence between Asian and European pigs, indicating that the MMS breed was the ancestral population of the Chinese pigs examined in our study. This could explain why there are still some ancestral haplotypes of MMS in current Chinese pig populations.

### Effective Population Size

**FIGURE 2:**
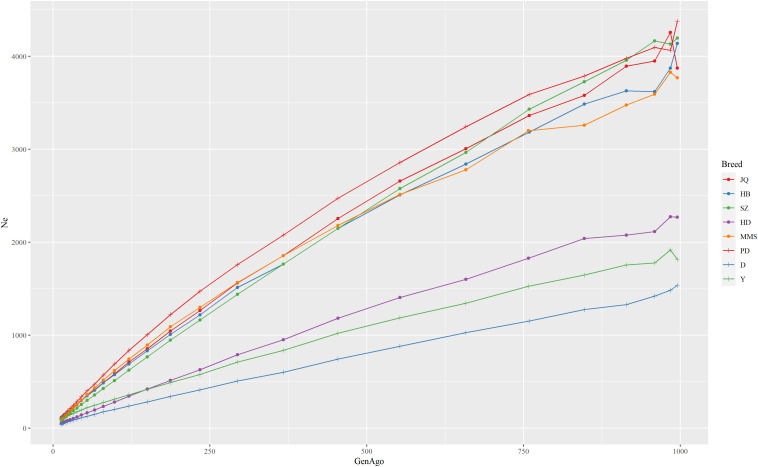
The estimate of the effective population size (*N*_*e*_) trend of each pig breed from 13 to about 1000 generations ago. The genome-wide estimate of *N*_*e*_ was based on the linkage disequilibrium between SNPs and corrected for sample size, mutation, and recombination rate. Each line shows the trend in effective population size across generations. The result showed that the JQ breed had experienced a rapid decline in its population, including the most recent generation.

The estimated *N*_*e*_ trend of each pig breed across different generations is shown in Figure 2. This estimate can improve our understanding of the demographic history of each population in the recent past (Barbato et al., 2015). While the extent of LD over longer recombination distances reflected more recent *N*_*e*_, that over shorter distances provided ancestral *N*_*e*_ (Hayes et al., 2003). The result showed that all the breeds had experienced a decrease in *Ne* estimate over time, especially from 900 to about 50 generations ago. We observed the nearest anti-climax points between 900 and 1000 generations ago, which indicated the nearest starting point of human-driven artificial selection that might have caused a population bottleneck in the breeds. In general, the western pig breeds had smaller *Ne* compared to the Chinese pigs and this can be attributed to the higher LD observed in western pig breeds (Amaral et al., 2008). In particular, we observed that the effective population size in the last 13 generations of the JQ breed was about 109 and about 3,871 in ∼1000 generations ago. This reduction might be due to an increase in inbreeding rate and a reduced genetic diversity usually observed in animals with a small population size (Food and Agriculture Organization, 2013).

### The Extent of Linkage Disequilibrium Across Breeds

A total of 268,369, 221,481, 313,100, 208,264, 202,599, 234,967, 171,952, and 219,248 autosomal SNPs were found in the JQ, HB, SZ, HD, MMS, PD, D and Y pig breeds, respectively. While, on the SSCX, we obtained 2,566, 2,416, 4,497, 2,013, 2,191, 2,995, 1,726, 2,709 SNPs in the respective breeds. These SNPs (on SSCX) were only utilized in characterizing the LD and haplotype block structure of the breeds.

**FIGURE 3:**
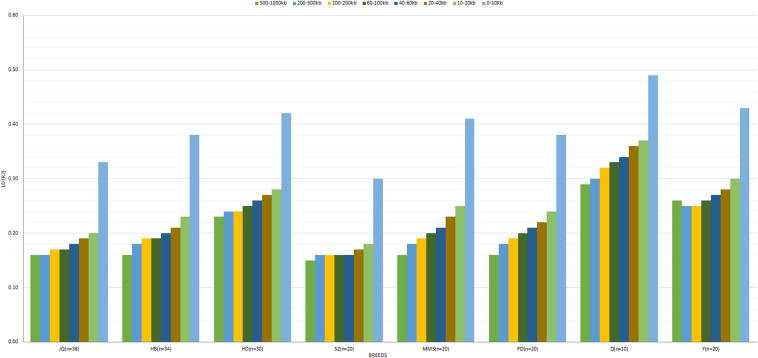
The averaged LD (*r*^2^) value at SNP distances 0–10, 10–20, 20–40, 40–60, 60–100, 100–200, 200–500, and 500–1000 kb across breeds. Jiangquhai (JQ), Huaibei (HB), Hongdenglong (HD), Shanzhu (SZ), MiddleMeishan (MMS), PudongWhite (PD), Duroc (D), and Yorkshire (Y). This LD estimate is non-fitted.

The average *r*^2^ between adjacent SNPs on the autosomes was largest for D breed (*r*^2^ = 0.39), followed by Y (*r*^2^ = 0.34), whereas other pigs exhibited a smaller average *r*^2^, ranging from 0.22 (SZ) to 0.32 (HD). The average autosomal LD (*r*^2^) for the following bin distances 0–10, 10–20, 20–40, 40–60, 60–100, 100–200, 200–500, and 500–1000, is presented in Figure 3. On the SSCX, the average *r*^2^ value observed for both D (*r*^2^ = 0.45) and Y (*r*^2^ = 0.37) breed was also the highest. However, the average LD (*r*^2^) decreased in other breeds; PD (*r*^2^ = 0.32), HD (*r*^2^ = 0.32), SZ (*r*^2^ = 0.31), MMS (*r*^2^ = 0.29), HB (*r*^2^ = 0.27), and JQ (*r*^2^ = 0.23). Overall, on the autosome of the JQ breed, about 28% of adjacent SNP pairs had *r*^2^ > 0.3 and 36% had *r*^2^ > 0.2. The corresponding percentages for HB, SZ, HD, MMS, PD, D, and Y were about 32 and 41%, 25 and 33%, 40 and 41%, 34 and 41%, 32 and 39%, 48 and 56%, 42 and 50%, respectively.

In general, the genome-wide average LD (*r*^2^) across breeds decreased with increasing SNP pair distance (Figure 3 and Supplementary Figure S1). A lower LD, which rapidly decayed with increasing genomic distance, especially for distances greater than 10 kb, was observed across breeds. As expected, large LD differences were observed between the Western (especially D) and Chinese breeds. Interestingly, we found that the LD decay on SSCX, across each breed, was slower compared to the autosomes’ (Supplementary Figure S1). We also observed that the LD decay on the SSCX of D breed was slower than other breeds, while that of the JQ breed was faster. Apart from D and Y breeds, the PD breed also had a slower LD decay on the SSCX compared to other breeds in the study.

### Haplotype Block Structure and Haplotype Diversity

**FIGURE 4:**
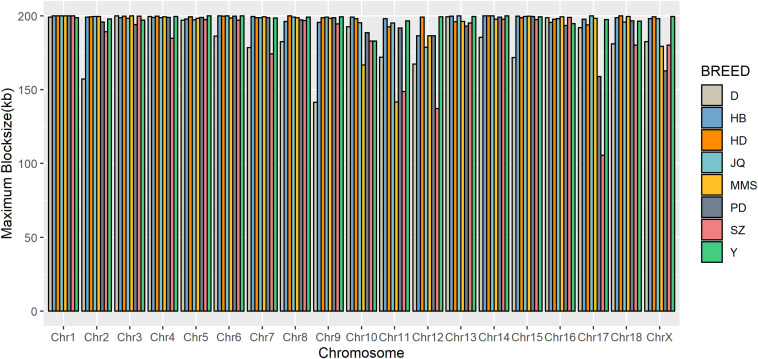
Distribution of maximum haplotype block length per chromosome across breeds. Jiangquhai (JQ) and Huaibei (HB) had more chromosomes with maximum block length compared to other breeds.

To gain insight into the systematic difference in the level of LD across each pig breeds, we characterized their haplotype blocks. Among all the pig breeds analyzed in this study, the JQ breed had the highest total autosomal block length, 345.30 Mb (14.18% of its total genome) while HB, SZ, HD, MMS, PD, D, and Y had 300.83 Mb (12.35%), 92.20 Mb (3.79%), 330.41 Mb (13.57%), 167.88 Mb (6.90%), 211.38 Mb (8.68%), 33.04 Mb (1.36%), and 176.35 Mb (7.24%) total autosomal block length, respectively. Moreover, fewer haplotype blocks (2,286) were observed on the autosome of D breed compared to others (Table 1), possibly due to a bias in its small sample size and a high percentage of fixed markers that were not involved in the haplotype block construction. On the SSCX, the total lengths of block (and average block size) were 4.43 Mb (15.32 kb), 6.49 Mb (23.09 kb), 2.75 Mb (12.31 kb), 5.61 Mb (21.50 kb), 2.62 Mb (22.75 kb), 1.96 Mb (14.52 kb), 1.51 Mb (47.05 kb), and 3.40 Mb (15.76 kb) for JQ, HB, SZ, HD, MMS, PD, D, and Y breed, respectively (Table 2 and Supplementary Tables S4–S10). We also found that the number of maximum haplotype block size per chromosome across breeds was larger in both JQ and HB except for SSC3, SSC4, SSC5, SSC8, SSC9, SSC12, SSC15, SSC16, SSC18, and SSCX (Figure 4).

**TABLE 1:** The number of haplotype blocks, haplotype frequency, and diversity across breeds.

Breed	JQ	HB	SZ	HD	MMS	PD	D	Y
No. of haplotype blocks	31146	26619	19362	25645	12312	14861	2286	16649
Haplotype frequency	0.25	0.26	0.34	0.27	0.29	0.29	0.36	0.30
Haplotype diversity	0.464	0.465	0.483	0.467	0.482	0.489	0.525	0.484

*Results in the table are derived from autosomal blocks.*

**TABLE 2:** Block statistics of JQ breed.

			Block size (kb)				SNPs (*n*)			
		Total block				No. of SNPs				% of SNPs
SSC	Blocks (*n*)	length (kb)	Mean	Min	Max	in blocks (*n*)	Mean	Min	Max	in blocks
1	2336	43176.07	18.48	0.002	199.95	10587	4.53	2	44	7.50
2	1908	28761.04	15.07	0.002	199.56	9326	4.89	2	52	6.61
3	2385	24841.31	10.42	0.002	198.34	10883	4.56	2	37	7.71
4	1741	18151.28	10.43	0.002	198.69	7621	4.38	2	40	5.40
5	1662	14397.77	8.66	0.002	197.18	7353	4.42	2	51	5.21
6	2838	34404.52	12.12	0.002	199.91	13751	4.85	2	55	9.75
7	2003	17262.21	8.62	0.002	198.72	8767	4.38	2	48	6.21
8	1330	13453.34	10.12	0.002	199.18	5594	4.21	2	32	3.96
9	1955	22753.54	11.64	0.002	199.19	9091	4.65	2	43	6.44
10	1398	8856.84	6.34	0.002	195.37	5995	4.29	2	35	4.25
11	997	9979.21	10.01	0.002	195.15	4327	4.34	2	27	3.07
12	1514	8364.55	5.53	0.002	178.76	6515	4.30	2	26	4.62
13	1681	22458.83	13.36	0.002	199.97	7277	4.33	2	41	5.16
14	2362	25559.27	10.82	0.002	199.91	10891	4.61	2	43	7.72
15	1688	24085.86	14.27	0.002	199.60	7493	4.44	2	33	5.31
16	1064	11134.99	10.47	0.002	198.01	4456	4.19	2	33	3.16
17	1268	10090.86	7.96	0.002	199.97	5647	4.45	2	55	4.00
18	1016	7565.22	7.45	0.002	195.75	4349	4.28	2	80	3.08
X	289	4427.64	15.32	0.002	198.11	1177	4.07	2	23	0.83
**Total**	**31435**	**349724.35**	**10.90**			**141100**				**100.00**

**FIGURE 5:**
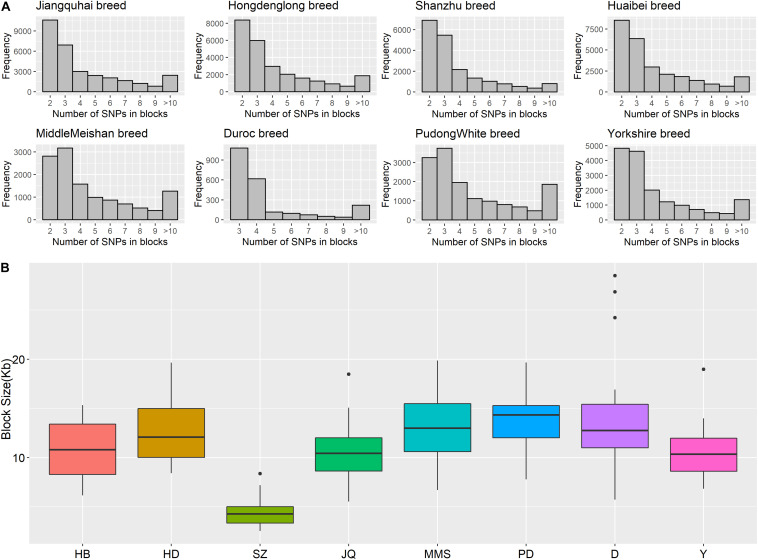
**(A)** Histogram plot showing SNP distribution in haplotype blocks across breeds. Jiangquhai (JQ) had more SNPs clustered into haplotype blocks than other breeds. **(B)** Box plot of haplotype block size distribution in different breeds. Shanzhu (SZ) had the shortest average haplotype block size while PudongWhite (PD) had the longest.

**FIGURE 6:**
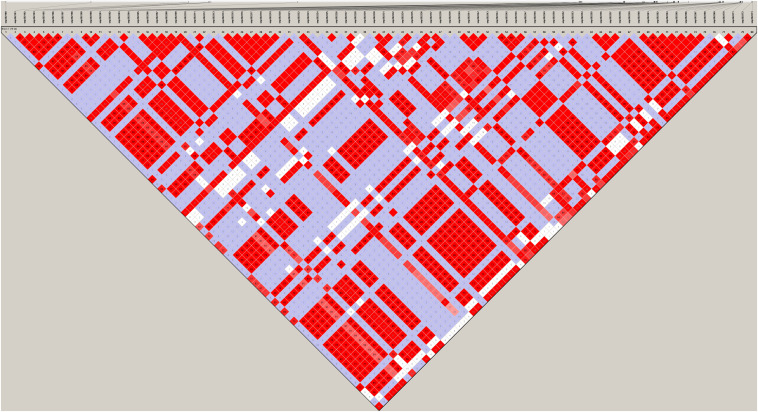
Haploview plot of linkage disequilibrium (*r*^2^) between SNPs on chromosome 18 of JQ breed. This block is 79.084 kb in size and has the maximum number of SNPs (80) in JQ blocks. Values in the diamond are LD values in percentages and diamonds without a value shows a complete LD (*r*^2^ = 1) between SNPs.

**FIGURE 7:**
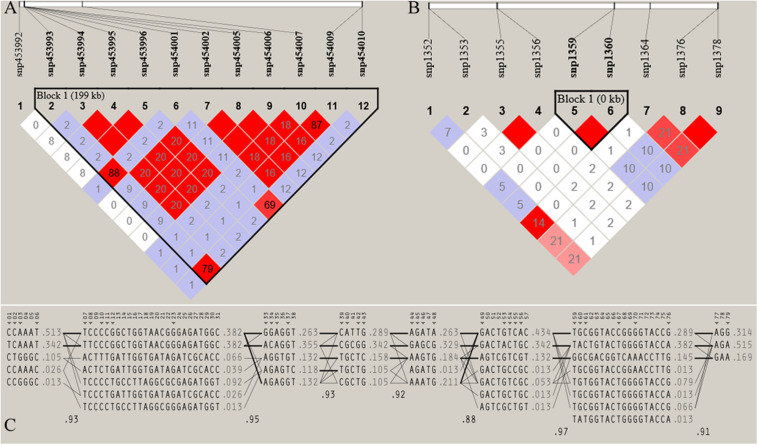
Haploview plot of linkage disequilibrium (*r*^2^) between SNPs located on **(A)** Chromosome 17 (42665098 bp – 42865069 bp) of JQ breed, with the maximum block length (199.97 kb) and **(B)** chromosome 1 (1936544 bp – 1936545 bp) of the same breed, with the minimum number of SNPs and block size (0.002 kb). This block overlapped the *UNC93A* gene **(C)** Haplotype block structure of chromosome 18 of JQ breed (with the Maximum number of SNPs (80snps). Marker numbers are shown across the top, with highlighted tag SNPs. The population frequencies of each haplotype are shown next to them with lines showing the most common crossings from one block to the next. The thicker lines indicate more common crossings than thinner lines and below the crossing lines is the multi-locus *D* prime between two blocks. Lower *D* prime value indicates a greater amount of historical recombination between two blocks (Barrett et al., 2005).

Furthermore, the coverage rate of blocks per chromosome in the JQ breed was higher than those of other breeds except for *Sus scrofa* chromosome 4 (SSC4), SSC6, SSC7, SSC8, SSC10, SSC12, SSC13, SSC14, SSC17, SSC18, and SSCX (Supplementary Table S11). The average block size in JQ breed was 10.90 kb (ranging from 0.002 to 199.97 kb) (Table 2). The average block size distribution across breeds is presented in Figure 5B. We also investigated the pattern of LD in the haplotype block region with the highest number of SNPs in the JQ breed. This block displayed a moderate LD (Figure 6) and high haplotype diversity (Figure 7C) suggesting that several recombination events might have occurred in this haplotype block. Furthermore, we observed a low LD level in the haplotype block with the maximum block length (199.97 kb), and a complete LD in the block with the minimum number of SNPs and block size (0.002 kb) (Figures 7A,B).

Generally, we found that the haplotype frequency and diversity across breeds (Table 1) were lower in all the Jiangsu pig breeds (JQ, HB and HD) except for SZ breed which exhibited a higher haplotype frequency and diversity similar to that of Y.

### Distribution of SNPs in Haplotype Blocks

The density of SNPs in the Chinese and western pig population in this study is presented in Supplementary Figures S2, S3. The summary of SNPs distribution and proportion involved in the haplotype block formation per chromosome across breeds was also presented in Table 2 and Supplementary Tables S4–S10. In summary, a total of 139,923, 116,377, 74,629, 113,762, 61,489, 77,729, 12,080, 75,997 SNPs located on the autosomes of JQ, HB, SZ, HD, MMS, PD, D, and Y breed were clustered into haplotype blocks respectively. These SNPs account for about 52.14, 52.55, 23.84, 54.62, 30.35, 33.08, 7.03, and 34.66% of all the autosomal SNPs in the respective breeds.

The frequency distribution of SNPs in the haplotype blocks for each breed is presented in Figure 5A. Generally, we observed a small proportion of haplotype blocks with more than 10 SNPs across each breed in this study. However, JQ and HD breeds had the highest number of blocks, with at least 10 SNPs. Intriguingly, among all the Chinese breeds in our study, the JQ breed had the highest number of SNPs in a block, with 80 SNPs in Block 22 of Chromosome 18 (923784 bp – 1002867 bp). This block overlaps the protein tyrosine phosphatase receptor (*PTPRN2*) gene, which suggests that it is associated with oncogenic processes (Bourgonje et al., 2016). This gene is also predominantly expressed in endocrine and neuronal cells, where it functions in exocytosis (Sorokin et al., 2015). Generally, JQ pigs are known for their high resistance to porcine reproductive and respiratory syndrome virus (PRRSV) infection (Meng et al., 2018).

This study also discovered that the highest amount of SNPs involved in block formation on chromosomes is observed in the JQ and other Jiangsu pig breeds (13,751, 12,403, 6,966, and 11,738 SNPs on Chromosome 6 of JQ, HB, SZ, and HD, respectively), while the lowest amount of SNPs on the autosomes of these pig breeds was 4,327, 3,083, and 3,013 on chromosome 11 of JQ, HB, and HD; and 2,276 on chromosome 16 of SZ breed. Conversely, the highest number of SNPs in other pigs was 7,940 and 6,274 on chromosome 6 of PD and MMS; 1,183 on chromosome 1 of D; and 7,799 on Chromosome 6 of Y breed. However, the lowest (total) number of SNPs in blocks (formed on the autosome) was 2,488, 1,580, 338, and 2,070, and was found on chromosome 16, 16, 18, and 16 of PD, MMS, D, and Y breed, respectively (Supplementary Tables S7–S10).

### Shared and Unique Haplotype Block Regions Between Breeds

As shown in Table 3, among all the pig breeds considered in this study, HD had the lowest percentage of shared haplotype blocks (with other breeds) while the SZ and MMS breed had the highest percentage of shared haplotype blocks. This result could be linked to the ancestral origin of MMS and the high admixture observed in SZ (Figure 1C). Among all the Chinese pig breeds in our study, the SZ breed had the highest percentage of shared haplotype block (18.12%) with Y (Table 3), indicating a high introgression of Y haplotype into the SZ breed. This result is also in line with our admixture analysis (Figure 1C) which suggests that the SZ breed might have been introgressed with different breeds in the past. JQ, HB, and PD breeds also shared a considerably high percentage of haplotype blocks with the western breeds (D and Y), suggesting an introgression between western pigs and these Chinese breeds. All the pig breeds included in our study, shared the highest percentage of their haplotype block with the JQ breed, which suggests a common ancestry (or introgression) between JQ, the western breeds (Bosse et al., 2014), and other Chinese pigs in our study. This could also be because the JQ breed had the highest number of haplotype blocks (Table 3).

**TABLE 3:** The percentage of common haplotypes shared across populations.

	JQ	HB	SZ	HD	MMS	PD	D	Y
**JQ**	**100**	27.81	12.09	25.86	18.73	22.17	2.60	13.00
**HB**	31.93	**100**	10.65	27.04	18.16	20.74	2.73	12.95
**SZ**	45.28	34.76	**100**	27.46	23.10	27.72	2.34	18.12
**HD**	27.03	24.62	7.66	**100**	16.43	18.28	2.36	12.00
**MMS**	38.52	32.55	12.69	32.34	**100**	31.12	2.44	12.46
**PD**	36.21	29.51	12.09	28.57	24.72	**100**	2.45	13.20
**D**	27.15	24.82	6.54	23.55	12.41	15.68	**100**	15.25
**Y**	25.44	22.09	9.48	22.48	11.86	15.82	2.86	**100**

*Each row represents the percentage of common haplotype blocks between the breed (on the first column) and other breeds across the row.*

In general, the total length of haplotype block shared across the autosome of all the Jiangsu province pigs (JQ, HB, SZ, and HD) was 7.89 Mb (Supplementary Data). These shared haplotype blocks could indicate the existence of conserved genomic regions that are a product of intensive and directional natural or artificial selection in the Jiangsu pig population. The plot of shared and unique haplotype block region in the Jiangsu pig population is presented in Supplementary Figures S4–S6.

### Functional Annotation of Overlapping QTLs and Genes

We detected the QTLs spanned by the haplotype block regions of JQ breed by finding the overlapping regions with 25,388 QTLs (length ≤ 10 Mb) downloaded from the pig QTL database. Consequently, 112 porcine QTLs were detected to overlap with the haplotype block regions of this breed. Interestingly, we found that most of the detected QTLs were related to meat and carcass quality, health, and a few reproduction and production-related QTLs. We detected QTLs related to traits such as feed conversion ratio, loin muscle area, body weight, intramuscular fat content, scrotal/inguinal hernia, teat number, total number born alive, change in *Mycoplasma hyopneumoniae* antibody titer, and toll-like receptor 9 level (Table 4), which suggest that the pig breed might have previously undergone selection for meat quality and health (an indication of the environmental adaptability of the breed). Specifically, about 13% of the QTLs (based on QTL IDs reported in the Pig QTL database), overlapping in the 20 scanned blocks in the JQ pig breed were related to drip loss (DRIPL) (water holding capacity of pork meat), and loin muscle area (LMA).

**TABLE 4:** QTLs associated with haplotype block regions in the JQ Breed.

SSC	No. of Haplotypes	Location (bp)	Size (kb)	No. of SNPs	QTLs
1	6	57754743–57954471	199.730	9	DRESS%, LMA, FEEDIN, AFR, SHEAR, FA-C20:1, FA-C18:0
1	17	100151813–100351758	199.946	26	DRIPL
1	17	145630575–145788505	157.931	44	–
2	23	150363020–150561606	198.587	52	LMA, BLACT
5	24	17274912–17439428	164.517	51	**–**
6	4	48308117–48507976	199.860	5	TOPLC, IHERN
6	8	52898408–53098319	199.912	5	TOPLC, FEEDIN
6	18	64203148–64347705	144.558	55	IMF, HAPT, C3C, NEUT, LEANWT, EBPC, FIRM, BFT, SHOUFATD, CTISSP, BFS, GLYPO, LMA, COOKL
6	27	65204715–65346283	141.569	41	LEANCUTP, LEANP, DRESS%
6	9	107929215–108129083	199.869	12	FAPC
7	20	57703699–57797277	93.579	48	TNUM
9	20	41012098–41114458	102.361	43	LVNUM, HDL
13	7	71557016–71756980	199.965	19	–
14	12	47714888–47914794	199.907	18	ANDR
14	13	49585363–49722310	136.948	43	TVNUM, CHOL
14	9	50940408–51140288	199.881	11	SCF, AGEP, RTNUM, TNUM, TNUMD
14	21	51322222–51522048	199.827	33	PLTCT
17	8	42665098–42865069	199.972	11	ACTH2, 34RIBBFT
17	13	62089019–62255688	166.670	55	pH
18	23	923784–1002867	79.084	80	MHT, MHTC, TLR9, DIAMF, FIB1DIAM, FIB2ADIAM, LIVWT, FEEDCON, ADG, BW, WWT, NBA, TNB

*List of full names of QTLs: DRESS%, dressing percentage; LMA, loin muscle area; FEEDIN, daily feed intake; AFR, average feeding rate; SHEAR, Shear force; FA-C20:1, *cis*-11-Eicosenoic acid content; FA-C18:0, stearic acid content; DRIPL, drip loss; BLACT, lactate level; TOPLC, top line conformation; IMF, intramuscular fat content; HAPT, haptoglobin concentration; C3C, C3c concentration; NEUT, neutrophil count; IHERN, scrotal/inguinal hernia; LEANWT, lean meat weight; EBPC, empty body protein content; FIRM, firmness; BFT, average backfat thickness; SHOUFATD, shoulder subcutaneous fat thickness; CTISSP, connective tissue protein; BFS, side fat thickness; GLYPO, average glycolytic potential; LMA, loin muscle area; COOKL, cooking loss; LEANCUTP, lean cuts percentage; LEANP, lean meat percentage; FAPC, fat area percentage in carcass; TNUM, teat number; LVNUM, lumbar vertebra number; HDL, HDL cholesterol; TVNUM, thoracic vertebra number; AGEP, age at puberty; TNUM, teat number; TNUMD, teat number, difference between sides; RTNUM, right teat number; PLTCT, platelet count; SCF, backfat between 3rd and 4th last ribs; ANDR, androstenone, laboratory; CHOL, cholesterol level in meat; ACTH2, post-stress ACTH level; 34RIBBFT, backfat thickness between 3rd and 4th rib; pH, pH 24 h post-mortem (loin); TLR9, toll-like receptor 9 level; DIAMF, diameter of muscle fibers; FIB1DIAM, diameter of type I muscle fibers; FIB2ADIAM, diameter of type IIa muscle fibers; BW, body weight (birth); FEEDCON, feed conversion ratio; LIVWT, liver weight; ADG, average daily gain; WWT, body weight (weaning); MHT, mycoplasma hyopneumoniae antibody titer; MHTC, change in mycoplasma hyopneumoniae antibody titer; TNB, litter size; NBA, total number born alive.*

Furthermore, we identified a total of 88 genes harbored by the selected haplotype blocks (Supplementary Table S12), of which only 4 were significantly enriched (*p-value* ≤ 0.05). These genes were significantly enriched in 2 GO terms and 2 KEGG pathways (Table 5) which were related to a variety of molecular functions linked to immunity. It is of note that two of the enriched genes (*ACVRL1* and *ACVR1B*) found on SSC5 were enriched (GO:0003840) in the molecular functional process (*p-value* ≤ 0.01) associated with activin receptor activity, type I. However, the other 2 genes (*GGT5* and *GGT1*) which are located on SSC14 were enriched in the molecular functional process gamma-glutamyltransferase activity (*p-value* ≤ 0.01). These 2 genes (*GGT5* and *GGT1*) were also enriched in the signaling pathway related to ssc00460: Cyanoamino acid metabolism, and ssc00430: Taurine and hypotaurine metabolism (*p-value* ≤ 0.02).

**TABLE 5:** Candidate Genes detected in the haplotype blocks of JQ pig population.

SSC	Haplotype block position (Mb)	ID	Term	*P*-value	Candidate Genes
5	17.275–17.439	GO:0016361	Activin receptor activity, type I	0.009	*ACVRL1*, *ACVR1B*
14	49.585–49.722	GO:0003840	Gamma-glutamyltransferase activity	0.009	*GGT5*, *GGT1*
		KEGG:ssc00460	Cyanoamino acid metabolism	0.011	*GGT5*, *GGT1*
		KEGG:ssc00430	Taurine and hypotaurine metabolism	0.017	*GGT5*, *GGT1*

## Discussion

The evolutionary history of some pigs in certain regions of developing countries like China is poorly understood. The emergence of new breeds or sub-populations is a result of natural (adaptation) or artificial selection and this selection pressure plays a major role in shaping the genetic architecture and gene pool of extensively raised livestock species (Amaral et al., 2008; Khanyile et al., 2015). Despite recent research on the JQ pig population, there is still a dearth of information on the genetic architecture of economically important breed traits. This study, as one of the first reports on the haplotype block structure in the JQ pig breed, aimed to reveal the effects of selection pressure on its genome. We characterized the pattern of LD in the genome of the breed and detected various QTLs and genes spanned by haplotype blocks. We compared most of our results with the ones obtained in three other breeds from the same province (region) (HB, SZ, and HD breeds); two from Shanghai province (MMS and PD); and two western breeds (D and Y). From this comparison, JQ showed a higher level of variation in block structure and the number of SNPs involved in the block formation.

### Overlapping QTLs and Genes Detected in the JQ Breed

Conservation of animal genetic resources from a global perspective, focuses not only on endangered breeds but also on those that are not well utilized. Locally adapted breeds are always at risk of extinction, particularly when local populations have a preference for imported breeds. Generally, only a small proportion of breeds, particularly in developing countries, are involved in planned genetic improvement programs that aim to ensure efficient and sustainable utilization of these breeds. Therefore, developing countries like China should ensure that commercial pig strains are developed, while also maintaining the genetic diversity within the purebred population.

In our study, we detected a high percentage of haplotype blocks overlapping QTLs related to meat and carcass quality, and a few related to health. This suggests that these haplotype blocks may be potentially associated with economic traits like side fat thickness, intramuscular fat content, average backfat thickness, cooking loss, meat firmness, lean meat weight, and response to *Mycoplasma hyopneumoniae* in the JQ breed (Table 4). Various studies have already reported most of these QTLs in the pig quantitative trait loci (QTL) database. For example, on SSC1, Stratz et al. (2018) reported a highly significant QTL for Dressing percentage (ID = 161054); on SSC6, Le et al. (2017) identified significant QTL for top line conformation (ID = 126140); Choi et al. (2010) detected highly significant QTL for lean meat weight (ID = 16910); Liu et al. (2008) detected significant QTL for average backfat thickness (ID = 5980); and Choi et al. (2011) also detected highly significant QTL for meat firmness (ID = 21367). On SSC17, Stratz et al. (2013) detected significant QTL for pH 24 hr post-mortem (loin) (ID = 21865), while on SSC18, Uddin et al. (2010) identified significant QTL for *Mycoplasma hyopneumoniae* antibody titer (ID = 12330) and changes in *Mycoplasma hyopneumoniae* antibody titer (ID = 12331). Generally, JQ pigs are excellent producers of quality meat, used in the production of Rugao ham, and characterized by large body size and high lean percentage (Toldrá et al., 2014). We believe that this result might aid the further genomic study of meat quality and health-related traits in the JQ breed.

In our gene enrichment analysis, we also detected 4 health-related genes involved in various molecular functions in JQ pigs. These include *ACVRL1*, *ACVR1B*, *GGT5*, and *GGT1* gene. The *ACVRL1* gene is a TGFb/BMP type I receptor that plays a key role in the regulation of endothelial cell proliferation and maintenance of vascular integrity (Tual-Chalot et al., 2014), while *ACVR1B* acts in a paracrine manner on skin epithelial cells to suppress tumorigenesis (Qiu et al., 2011). These two genes also play essential roles in bone growth and morphogenesis (Merino et al., 1999), suggesting a pleiotropic SNP in the haplotype block region harboring these genes (Solovieff et al., 2013; Zhang et al., 2016). Furthermore, *GGT5* has been found to code for a cell surface protein that helps in the hydrolysis of the gamma-glutamyl bond of glutathione and glutathione *S*-conjugates (Wickham et al., 2011). It is expressed by macrophages throughout the body and may play an important role in the immune system (Hanigan et al., 2015). An increase in the expression of *GGT5* has also been found to impair testicular steroidogenesis by deregulating local oxidative stress (Li et al., 2016). The *GGT1* gene plays a major role in cleaving glutathione and its conjugate (Hanigan et al., 2015). In our study, *GGT5* and *GGT1* genes were also found to be enriched in the KEGG term (KEGG: ssc00430) related to Taurine and hypotaurine metabolism. Taurine is known to affect the cholesterol level in the body and can be found in various meat products (Laidlaw et al., 1990; Woollard and Indyk, 1993; Wójcik et al., 2010; Ripps and Shen, 2012). Interestingly, we found that this genomic region (Chromosome 14: 49585363 bp – 49722310 bp) overlapped the QTL that is suggestively associated with cholesterol levels in meat (CHOL) (Table 4). This suggests an association of this haplotype block with some meat quality traits in the JQ breed.

Although the haplotype block with the highest number of SNPs in this study was found within the Protein Tyrosine Phosphatase Receptor Type N2 (*PTPRN2*) gene in the JQ breed, to our surprise, it was not significantly enriched in any pathway or ontology. We infer that there could be more health-related genes in this block region (Figure 6) that are yet to be annotated as there were a lot of health-related QTLs, like Mycoplasma hyopneumoniae antibody titer (MHT), Change in Mycoplasma hyopneumoniae antibody titer (MHTC), and Toll-like receptor 9 level (TLR9), spanned by the block (Table 4). A previous study had investigated the degree of resistance to *M*. *hyopneumoniae* in JQ porcine lean strain (JQHPL) and concluded that JQHPL pigs exhibited higher resistance to *M*. *hyopneumoniae* than the western strains in the study, possibly due to the faster and stronger mucosal immunity phenotype of the strain (Hua et al., 2014). However, we also premise that this haplotype block could be harboring some disease susceptibility traits in the JQ breed, as the average MAF of its SNPs was about 0.20 (ranging from 0.07 to 0.5) (Supplementary Table S13). Generally, common variants (MAF > 5%) have been found to contribute to complex diseases more than rare variants (MAF < 1%) (Gibson, 2012; Bomba et al., 2017). Therefore, further study of this genomic region could help in understanding the genomic architecture of complex diseases in the JQ breed and also prevent the incorporation of such haplotype block into selection programs.

In a bid to establish a relationship between the candidate genes and QTLs detected in haplotype blocks, we linked the genomic regions of the detected candidate genes to the corresponding QTL region. Surprisingly, we found that only one gene overlapped these QTL regions. This is because the length of each QTL in these regions is greater than 10 Mb (ranging from 33.11 to 131.16 Mb). We premise this observation on the filtering of all QTLs with length > 10 Mb during our QTL annotation. However, we made some compromises (in QTL length) to enable us to have an overview of the quantitative traits in these genomic regions. In summary, *ACVRL1* and *ACVR1B* gene overlapped QTLs linked to CD4-positive leukocyte percentage (CD4LP), C3c concentration (C3C), and Hemolytic complement activity (alternative pathway) (AH50), while, *GGT5* and *GGT1* gene overlapped QTLs linked to Interferon-gamma to interleukin-10 ratio (IFNGIL10), Calcium level (BCAL), Creatinine level (CREAT), Potassium level (BPOTASS), C3c concentration (C3C), Haptoglobin concentration (HAPT), and Melanoma susceptibility (MELAN). This suggests an association of the haplotype blocks in these genomic regions with health-related traits in the JQ breed.

### The Extent of Linkage Disequilibrium in the Pigs and Application in GWAS

A full understanding of the LD properties in domesticated animals like pigs is of importance because it underlies all forms of genetic mapping (Nordborg and Tavaré, 2002) and can be used for fine mapping genes associated with complex diseases in pigs. To increase the power of SNP-based association studies (GWAS), the extent of LD in a breed must be considered. A knowledge of this can be used to predict the average number of markers required in quantitative trait association studies (GWAS).

In this study, we looked at the extent of LD in the JQ breed and compared it to the one obtained in other breeds. We used *r*^2^ value as a measure of LD between each locus of a chromosome. Generally, we observed a lower LD level, at larger SNP distances, on both autosomes and SSCX of JQ breed. A similar result was also found in the SZ breed (Figure 3 and Supplementary Figure S1). However, our result contradicts that of Xu et al. (2019), which reported a higher LD extent greater than 0.3, at SNPs distance of 99.66 kb in the JQ breed using Porcine 80 K SNP chips. This difference might be due to the larger sample size, density, and type of SNP data used in the study (as reviewed by Qanbari, 2020). Generally, SNP chips (or genotyping array) data tends to underrepresent rare variants that are likely to be detected in sequence data like the one used in this study. Since the extent of LD (*r*^2^) depends on MAF, it is expected that there would be a little difference in the *r*^2^ value obtained from both studies, partly due to SNP ascertainment bias on SNP chip data (Lachance and Tishkoff, 2013; Geibel et al., 2019). This kind of bias was reduced in a recent study by Huang et al. (2020) which had more Chinese breeds represented in the design of the SNP array used. The study reported an LD (*r*^2^ > 0.3) at SNPs-distance of 36.10 kb for the JQ breed. Moreover, using the MUC4 (Mucin 4, Cell Surface Associated) gene sequences, Yang et al. (2012) also reported that *r*^2^ > 0.3 extended up to 20 kb distance in the JQ breed, validating to some extent, the reliability of the LD value obtained in our study.

To assess the differences in LD extent on the autosome and SSCX across breeds, we predicted the extent of LD decay for different genomic distances. The result showed a noticeable difference in the LD extent across breed, especially on the SSCX (Supplementary Figure S1). As expected, we observed a longer LD extent on the SSCX compared to the autosome (Schaffner, 2004). Generally, the SSCX is known to have a low recombination rate and tends to preserve demographic events longer than the autosome (Schaffner, 2004; Laan et al., 2005). Among the Chinese pigs in this study, we observed a longer LD extent on the SSCX of the PD breed, suggesting that this breed might have recently evolved or experienced a bottleneck. This result is also in line with our admixture analysis which had the lowest cross-validation error when *K* = 7 (Figure 1B), before PD separated from MMS into a different cluster (Figure 1C). This result might also be useful in mapping sex-related traits in the Chinese pigs in our study.

According to previous studies, a mean *r*^2^ ≥ 0.3 is considered as a strong LD sufficient for QTL mapping (Farnir et al., 2000). However, to detect a QTL in GWAS and estimate the genomic breeding value (GEBV) of an animal, an average *r*^2^ of at least 0.2 is required to achieve power and accuracy ≥ 0.8 (Meuwissen et al., 2001; Meuwissen et al., 2001). In our study, we found that moderate LD (*r*^2^ ≥ 0.2) extended up to 500–1000 kb in HD (0.23), D (0.29), and Y (0.26) breeds (Figure 3). This suggests that an association study performed within these breeds using an average inter-marker *r*^2^ ≥ 0.20 would require about 12,000 SNPs. Although the average *r*^2^ (for bin distance 500–1000 kb), reported for the D and Y in our study was higher compared to the one reported by Grossi et al. (2017) using a 60 K SNP panel (0.23 and 0.17 for D and Y, respectively), we found that the average *r*^2^ for the autosomes of these breeds is still comparable to a previous study that used larger sample sizes (>100) (Badke et al., 2012). These differences in LD could be attributed to population structure, selection, sample size or density of the markers used in the study. On the other hand, *r*^2^ ≥ 0.20 extended only up to 0–10 kb in the SZ breed, and 10–20 kb in JQ (Figure 3), indicating that about 120,000 to 240,000 SNPs would be required for effective GWAS in these breeds. For the HB breed, the average inter-marker *r*^2^ extended up to 40–60 kb, meaning that about 40,000 to 60,000 SNPs would be needed for GWAS. While for MMS and PD, *r*^2^ extended up to 60–100 kb distances, and about 24,000–40,000 evenly spaced SNPs would be sufficient for a successful association study in the breeds. This result could be particularly useful in designing breed-specific SNP array panels for future genomic study and selection programs for these pig breeds.

### Haplotype Block Structure

The ability of an animal to survive in a changing environment, and also keep up with changes in selection preference, depends on the richness (genetic diversity) of its gene pool. This can be affected by several occurrences, including natural and artificial selection. There are various parameters for measuring genetic diversity in a population, including population-gene-frequency based statistics like average expected heterozygosity (He), the proportion of polymorphic loci (Pn), and allelic richness (Ar). However, alternative statistics based on allelic diversity (i.e., number of different allele types present at a locus) can also provide insight into the genetic diversity in a population and be a better predictor of long-term adaptation and total response to selection in an unpredictable future scenario (Caballero and García-Dorado, 2013; Vilas et al., 2015). Therefore, our study examined haplotype diversity as a measure of genetic diversity across breeds, since haplotypes are multi-allelic markers and can be treated as an allele in a haplotype-based study. To our surprise, we found that D pigs had higher haplotype diversity than the other Chinese pig breeds in this study (Table 1) despite having experienced high selection pressure in the past. Interestingly, among the Jiangsu province pigs in our study, the SZ breed had the highest haplotype diversity compared to JQ, HB, and HD (Table 1). Its haplotype diversity (0.483) (Table 1) was similar to that of Y with which it shared the highest haplotype block (18.12%) in comparison with other breeds (Table 3). This result is in agreement with our previous research (Zhang et al., 2018), which suggested that Y might have been used to improve the SZ breed and that the SZ breed might have originated from different genomic sources, therefore increasing the diversity of haplotypes in the breed’s genome. Furthermore, the higher haplotype diversity observed in the highly selected western pig breeds in our study could be because the sample size was selected from a larger population of western breeds (more than 1,000) in China, and a limited population of Jiangsu pig breeds (about 140 individuals) kept in the conservation pig farm in Jiangsu province, China. Therefore, we can also infer that the diversity of haplotypes in a population can be influenced by its population size. Moreover, recent studies had reported ongoing selection processes in Chinese pig breeds (Quan et al., 2020), which calls for the strategic management of these breeds to prevent the loss of important traits or genes. Although many studies had reported high genetic diversity in the JQ breed (Fan et al., 2002; Xu et al., 2019), most of these reported metrics do not perfectly reflect the ability of the breed to cope with a future unexpected change in breeding preference or disease outbreak. Therefore, the lower haplotype diversity observed in the JQ breed could be an indicator of a reduction in their genetic diversity, indicating a need for proper management of the JQ population in conservation pig farming. This result is also in line with the findings of Quan et al. (2020), which reported a lower haplotype diversity (0.752) in the mtDNA sequences of the JQ breed compared to that of Duroc (0.794), Yorkshire (0.837), and Meishan pigs (0.811). In line with our admixture and haplotype sharing result (Figure 1C and Table 3), the conservation farms could design breeding programs that restrict the level of contribution of the highly admixed JQ individuals to the next generation of the breed.

Furthermore, our block analysis revealed that the JQ breed had more SNPs that were clustered into haplotype blocks than any other breed in this study (Figure 5A), an indication that the breed contains a lot of variants that are inherited in the form of haplotype blocks. This could improve the fine mapping of QTLs and association studies in this indigenous pig breed. Our result also revealed that the JQ breed had a moderate block size (Figure 5B), which implies that the breed could have undergone moderate selection. This is also in line with the extent of LD observed in the breed (Figure 3). Besides, the smaller average haplotype block size observed in the SZ breed could be the result of historical admixture with western pig breeds, which is strongly supported by our Neighbor-Net tree (Figure 1A), admixture analysis (Figure 1C), and haplotype block sharing result (Table 3). Although our results showed variations in haplotype block structure across breeds, we were unable to detect some known haplotype blocks on the SSCX of each breed (Reimer et al., 2018) (shown in Supplementary Figure S7). This might be because the density of the SNPs on the SSCX (Supplementary Figures S2, S3) was not enough to track these SNPs and the resulting haplotype blocks.

The variation in the number of unique haplotype blocks within a population can shed more light on the independent genomic sub-structuring and evolution of such populations (Khanyile et al., 2015). Moreover, haplotype sharing allows the generational transfer of genomic materials between breeds (Khanyile et al., 2015). Our shared and unique haplotype block result showed different variations across breeds and reveals the level of uniqueness of each breed in our study. To strategically manage a pig population for conservation purposes, it is necessary to perform a SWOT (Strength, Weakness, Opportunity, and Threat) analysis of each breed. The strengths of a breed might be, for example, its genetic uniqueness, its adaptation to a particular system of production, or its past and present function in human culture (Food and Agriculture Organization, 2013). JQ pigs are generally well adapted to their local environment while they serve as a major source of meat for ham production in China (Toldrá et al., 2014). Breed-specific haplotype blocks in this pig could be considered a useful tool in characterizing and protecting its genetic diversity, as they potentially indicate a genomic source of unique phenotypic characters in the breed. Consistent with our admixture result (Figure 1C), a higher percentage of JQ blocks was (found in or) shared with D (Table 3), suggesting that the JQ breed might have experienced high introgression from D breed. This result is also in line with the known history of the breed. Generally, Chinese pigs, despite their superior meat quality and high prolificacy, have a slower growth rate compared to western pig breeds, and local pig farmers tend to supplement this by crossing indigenous pig breeds with commercial lines. However, if this is not properly managed in the JQ population, it might lead to a complete genetic erosion in the breed and also minimize their natural ability to adapt to local environmental stresses and disease outbreak.

The present study confirms previous findings (Fan et al., 2002; Xiao et al., 2017b; Xu et al., 2019) and contributes additional evidence that suggests that the JQ breed is indeed an important genetic resource. However, continuous genetic erosion and decline in the population, increases the risk of losing some economically important traits of the breed. We believe our result has provided more information that could guide the development of breeding programs to ensure the conservation and utilization of this genetic resource. In addition, the SZ breed showed the highest level of introgression from the Y breed and might be at the point of losing its genetic uniqueness. This indicates the weakness of this breed and should be taken into consideration when planning conservation programs in the future.

## Conclusion

We analyzed the LD and haplotype block structure of the JQ pig breed and also detected some underlying QTLs and genes spanned by these blocks. The present study revealed some blocks that might be associated with some quantitative or adaptive traits of the JQ pig and also provides useful information that could contribute to more informed, strategic management decisions in conserving and utilizing this breed. This result might also be useful in selecting variants for further association studies of these traits. We also reported a high level of introgression of Y haplotypes into the SZ pig breed and concluded that the later breed might be at the point of losing its genetic uniqueness.

## Data Availability Statement

All SNP data in the present study can be found in the FigShare Repository: 10.6084/m9.figshare.11984010.

## Ethics Statement

The animal study was reviewed and approved by the Institutional Animal Care and Use Committee of Shanghai Jiao Tong University (contract no. 2011-0033).

## Author Contributions

YP and QW conceived and supervised the study. FO analyzed the data and wrote the manuscript while other authors helped FO during the analysis and also revised the manuscript. All the authors revised and approved the manuscript.

## Conflict of Interest

The authors declare that the research was conducted in the absence of any commercial or financial relationships that could be construed as a potential conflict of interest.

## Footnotes

1 http://klab.sjtu.edu.cn/GGRS/

2 http://clumpak.tau.ac.il/distruct.html

3 http://www.r-project.org/

4 https://www.animalgenome.org/cgi-bin/QTLdb/SS/index

5 http://asia.ensembl.org/Sus_scrofa/Info/Index

6 https://david.ncifcrf.gov/home.jsp
