# Supplementary material for: Haplotype Block Analysis Reveals Candidate Genes and QTLs for Meat Quality and Disease Resistance in Chinese Jiangquhai Pig Breed
Source: Front Genet. 2020 Sep 4;11:752. doi: 10.3389/fgene.2020.00752 (PMC7498712; doi:10.3389/fgene.2020.00752)
Supplement: Supplementary file 1 [file Data_Sheet_1.docx]

Supplementary Material

1. **Supplementary Figures**
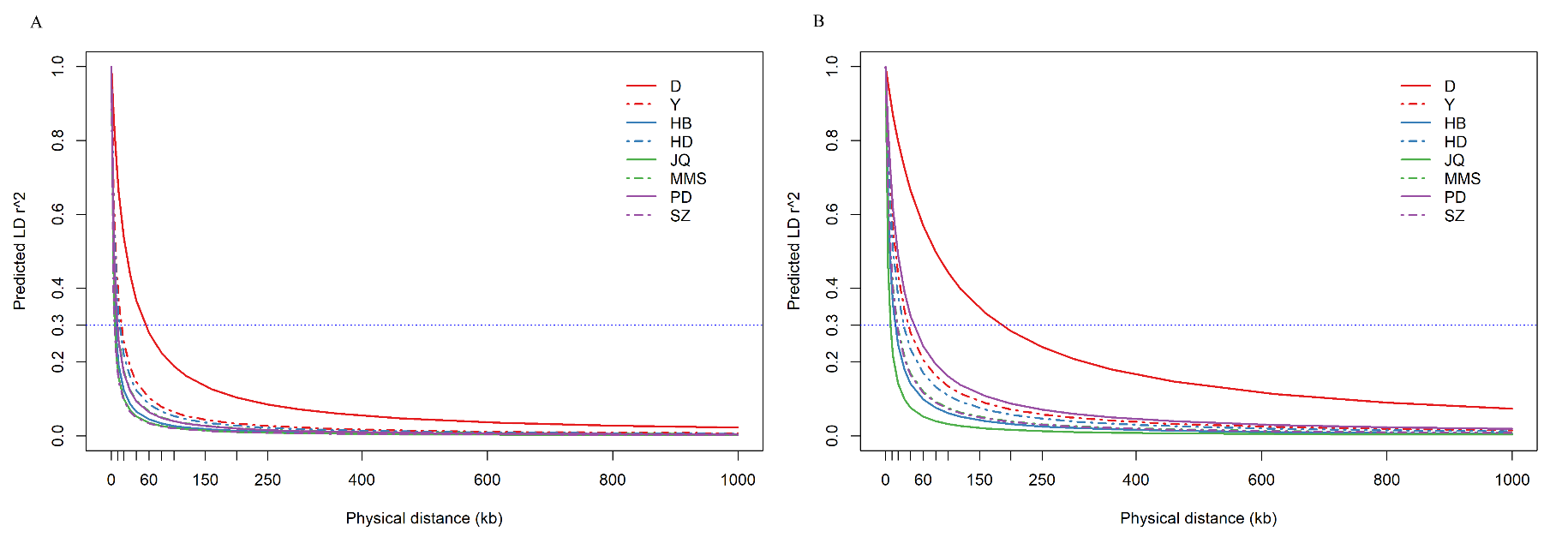


**Figure S1***.* Extent of LD on the (A)Autosome and (B) X-chromosome (SSCX) across breeds. The decay of LD (r2) values are plotted as a function of pair-wise inter-marker distances across breeds. The LD is fitted as mean LD for different bin distances, (indicated in the materials and methods section), on each chromosome of a breed.


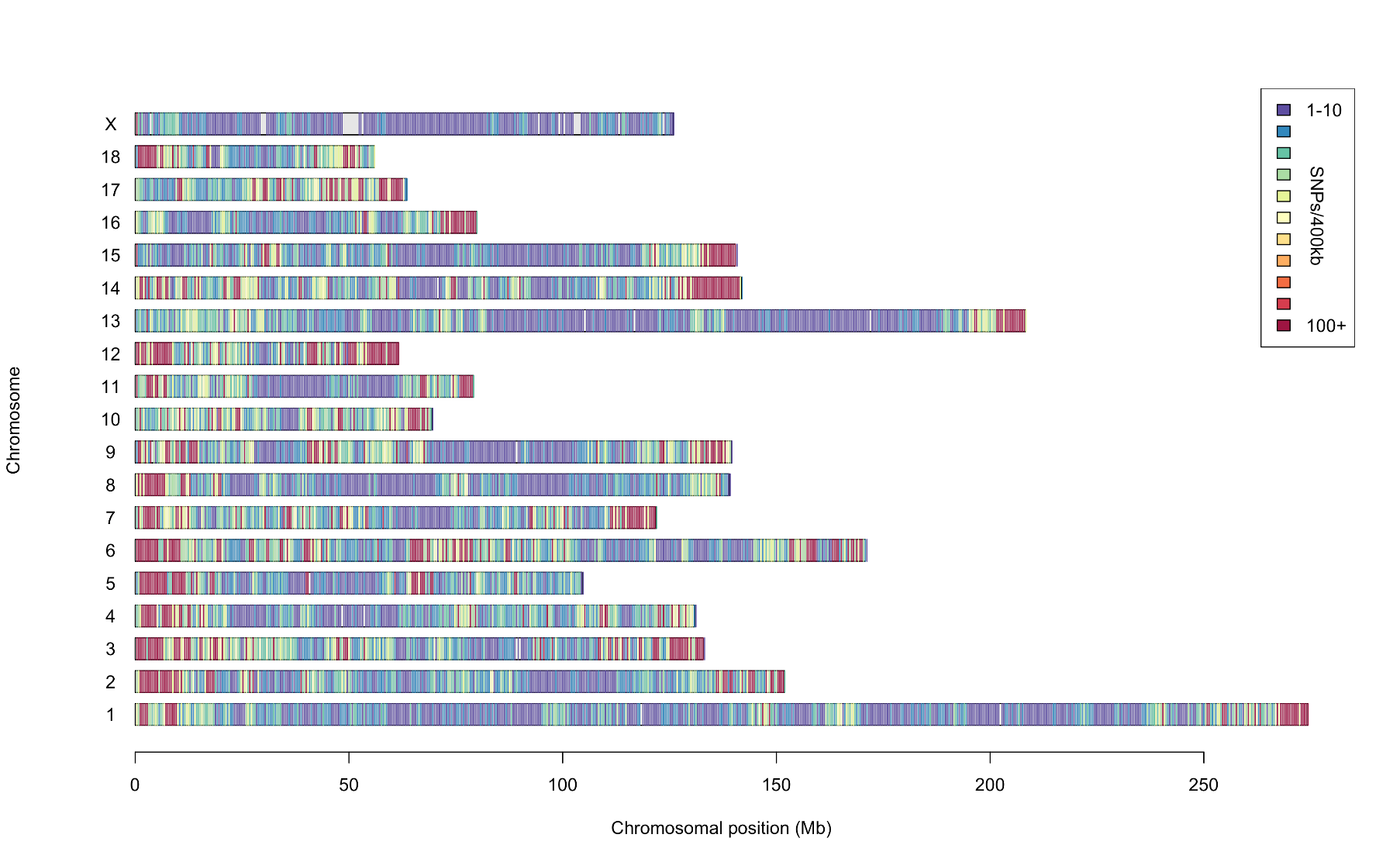
**Figure S2**. Distribution of 316,665 SNPs found in the combined dataset of Chinese pig population without removing in-breed monomorphic markers. The horizontal axis shows chromosome length (Mb); the different colors depict SNP density i.e the number of SNPs per 400kb window size.


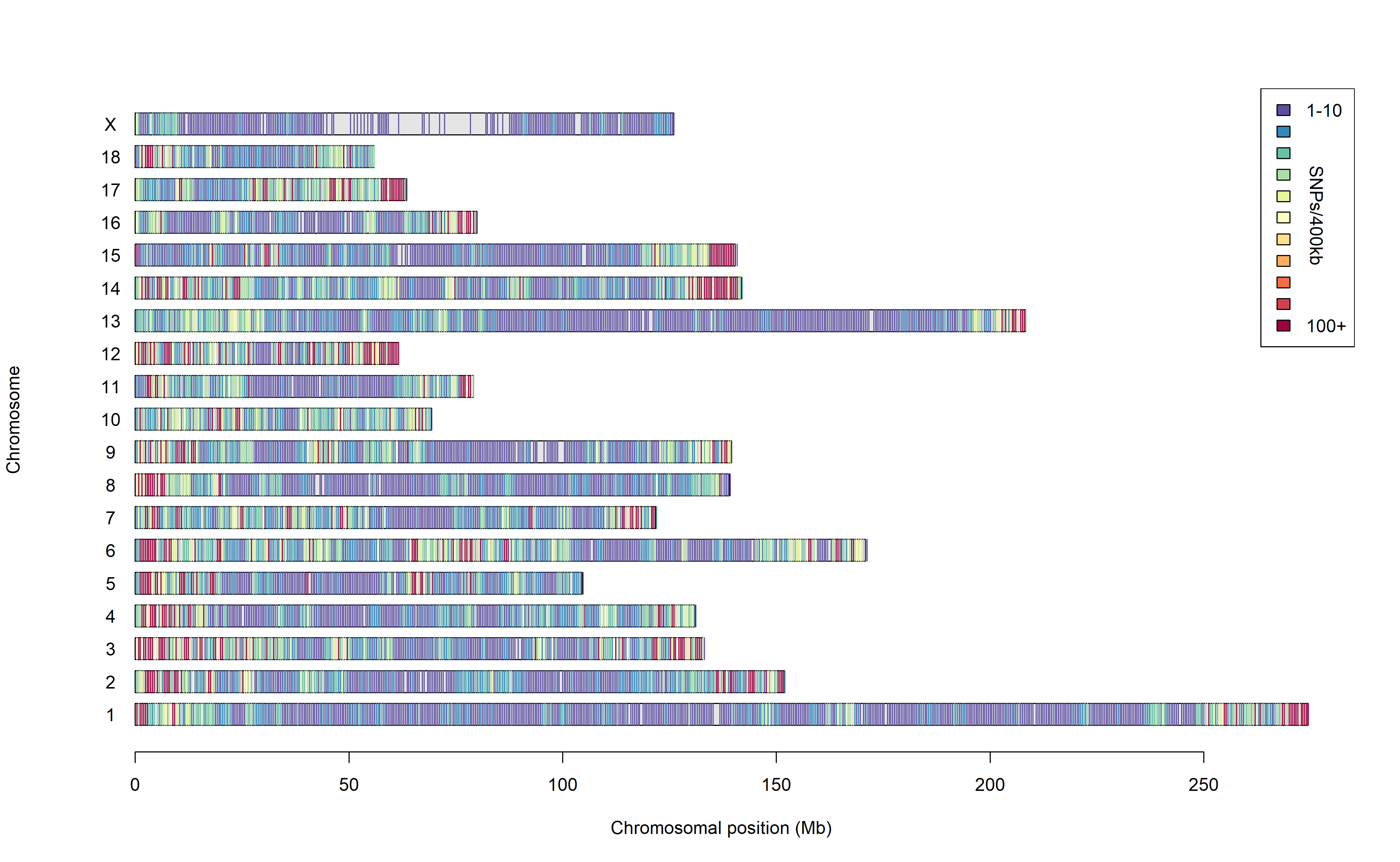
**Figure S3**. Distribution of 242,968 SNPs found in the combined dataset of Western pig breeds.


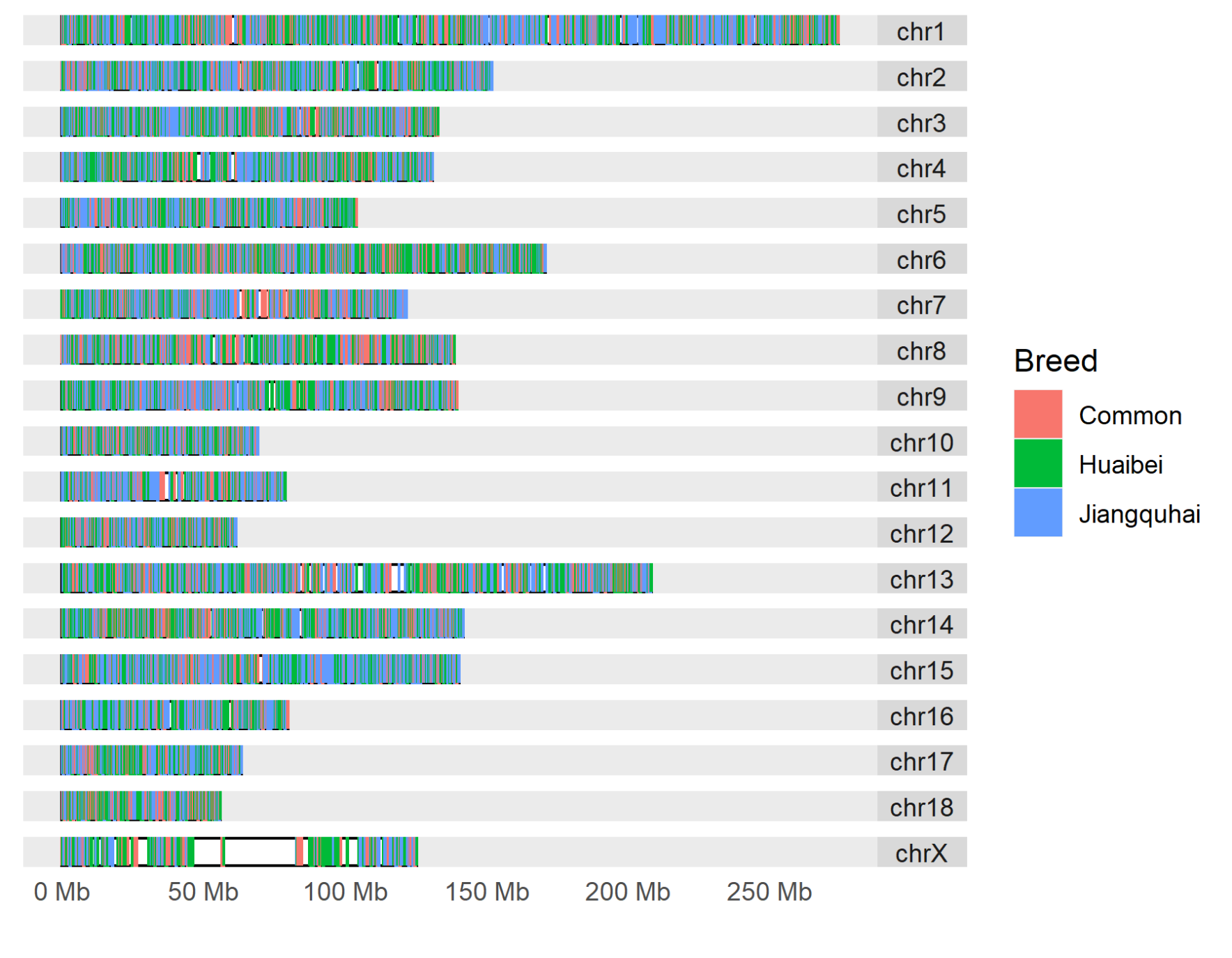


**Figure S4**. Shared and unique haplotype blocks between JQ and HB breed


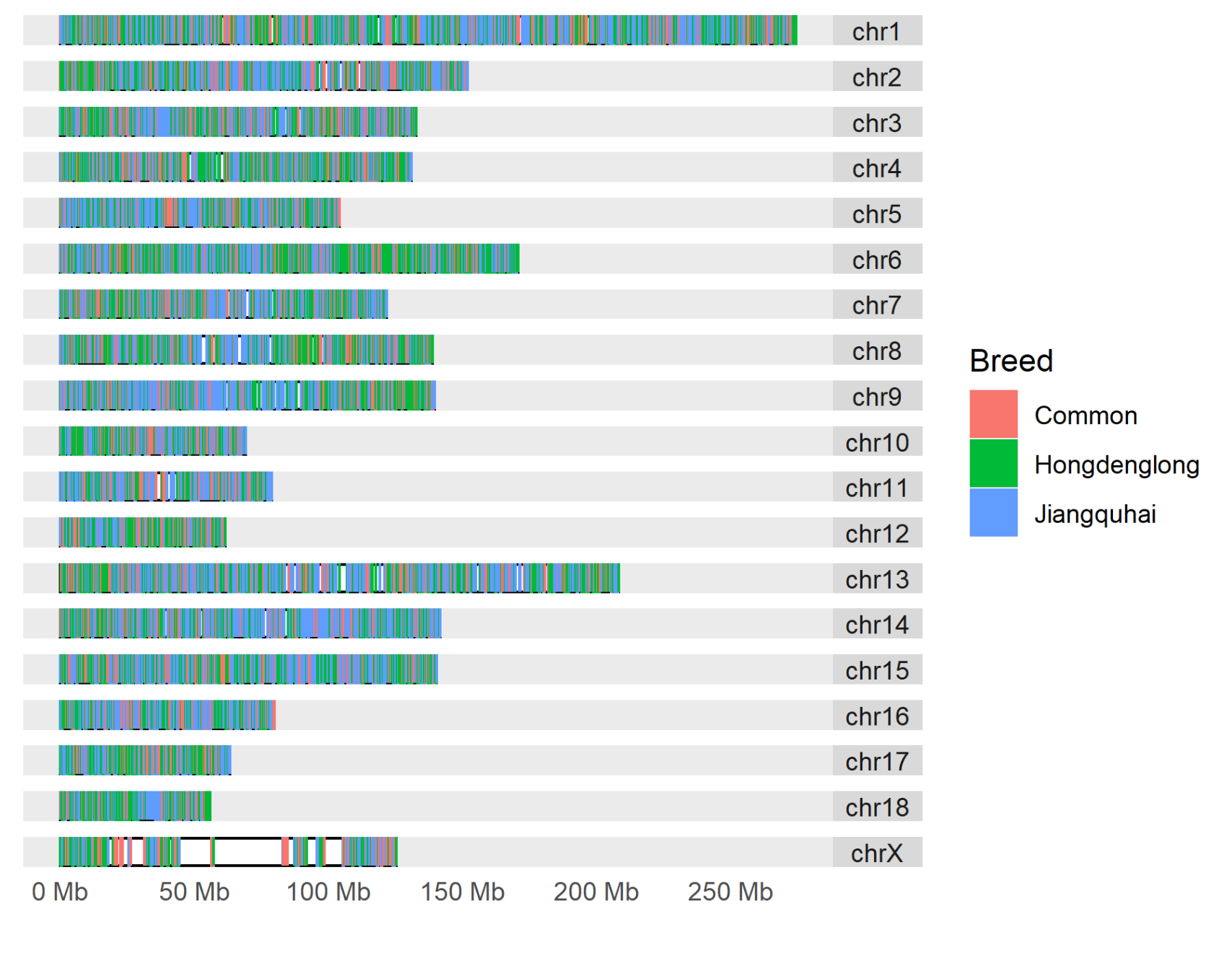


**Figure S5.** Shared and unique haplotype blocks between JQ and HD breed


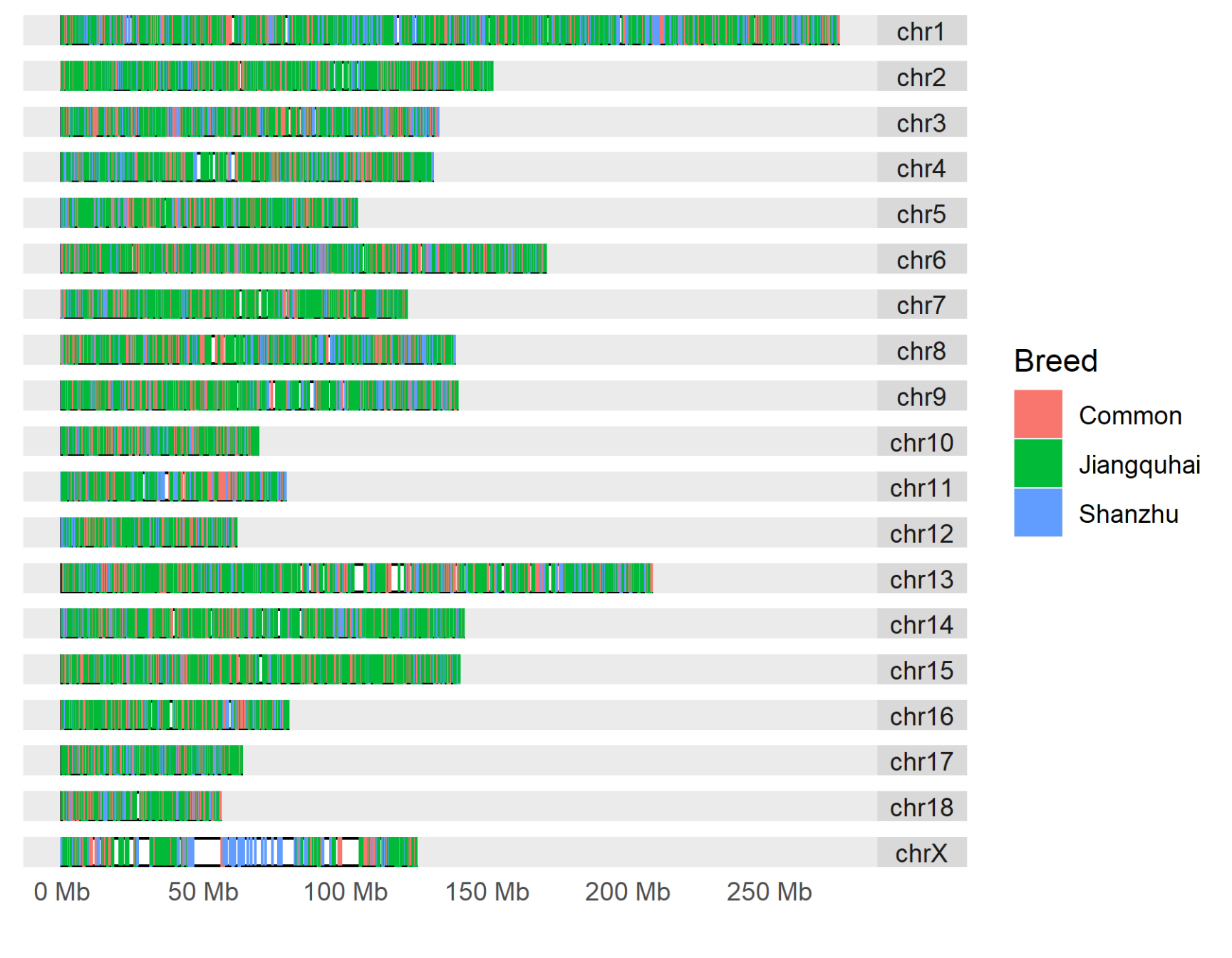


**Figure S6**. Shared and unique haplotype blocks between JQ and SZ breed


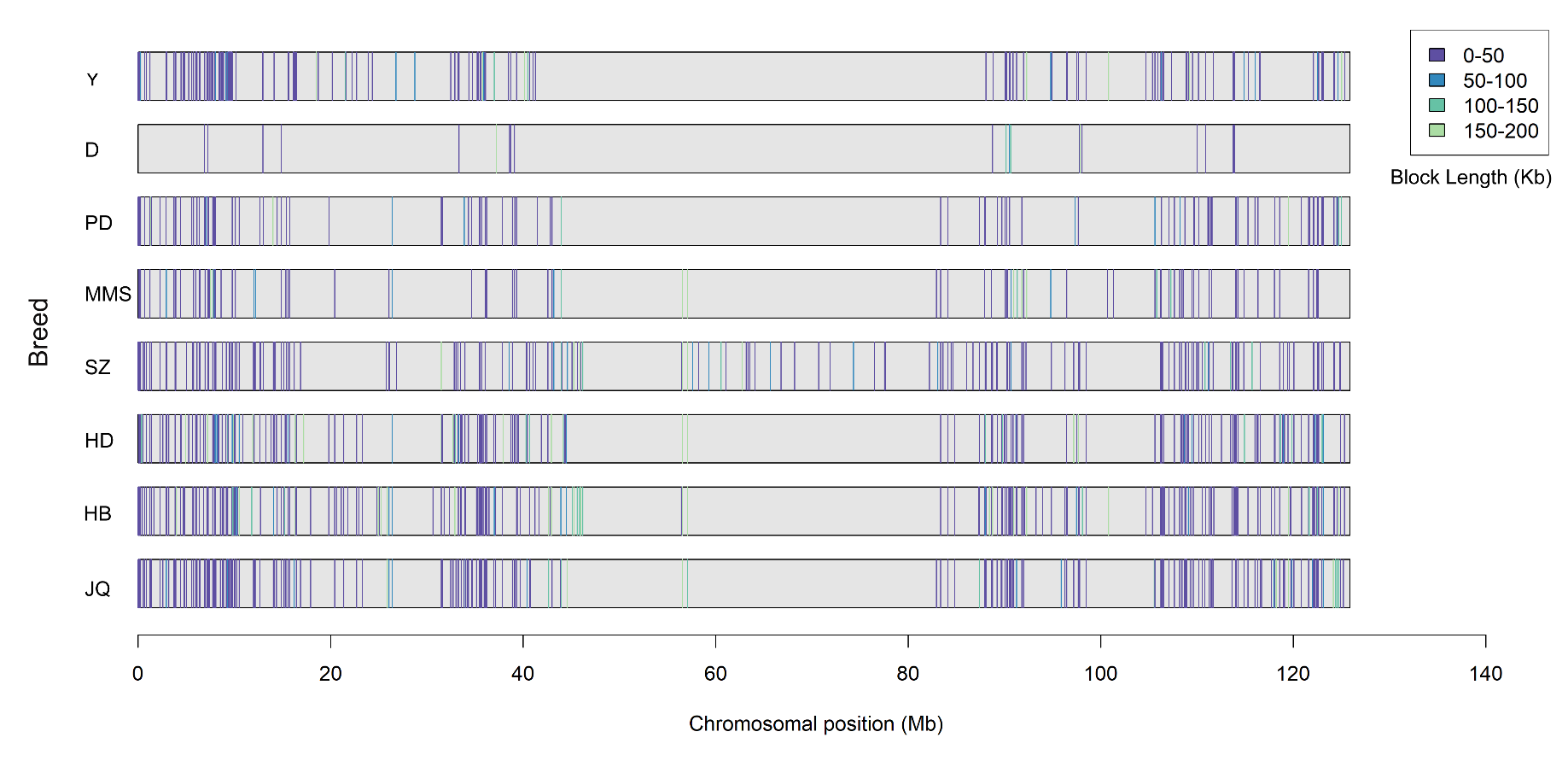
**Figure S7**. Distribution of haplotype blocks on the Chromosome X across breeds.

1. **Supplementary Tables**

**Table S1.** Alignment Statistics

| **Breed** | **Sample size** | **Average genome coverage (%)** | **Average sequencing depth^1^** |
| --- | --- | --- | --- |
| JQ | 38 | 2.7 | 10.18 |
| HB | 34 | 1.9 | 12.42 |
| SZ | 20 | 2.3 | 13.06 |
| HD | 30 | 2.5 | 13.26 |
| MMS | 20 | 1.8 | 12.29 |
| PD | 20 | 1.7 | 29.87 |
| D | 10 | 3.6 | 12.08 |
| Y | 20 | 2.8 | 10.72 |

^1^The sequencing depth was estimated based on the SNP identification

**Table S2.** Variant calling Statistics

| **Population** | **Total no. of SNPs** | **SNPs**  **(QS>20; DP>5X; MAF≥0.03)** | **SNPs^*^**  **(MAF≥0.05)** |
| --- | --- | --- | --- |
| **JQ** | 11,205,406 | 486,018 | 270,935 |
| **HB** | 11,205,406 | 486,018 | 223,897 |
| **SZ** | 11,205,406 | 486,018 | 317,597 |
| **HD** | 11,205,406 | 486,018 | 210,277 |
| **MMS** | 11,205,406 | 486,018 | 204,790 |
| **PD** | 11,205,406 | 486,018 | 237,962 |
| **D** | 11,205,406 | 486,018 | 173,678 |
| **Y** | 11,205,406 | 486,018 | 221,957 |

*Retained SNPs after filtering for MAF in the individual breeds, and discarding SNPs on the Y Chromosome.

**Table S3.** Genetic differentiation between populations measured by F_ST_ values.

| **Breeds** | **JQ** | **HD** | **SZ** | **HB** | **MMS** | **PD** | **D** | **Y** |
| --- | --- | --- | --- | --- | --- | --- | --- | --- |
| **JQ** | 0.000 |  |  |  |  |  |  |  |
| **HD** | 0.201 | 0.000 |  |  |  |  |  |  |
| **SZ** | 0.114 | 0.210 | 0.000 |  |  |  |  |  |
| **HB** | 0.157 | 0.243 | 0.146 | 0.000 |  |  |  |  |
| **MMS** | 0.115 | 0.203 | 0.151 | 0.160 | 0.000 |  |  |  |
| **PD** | 0.115 | 0.216 | 0.124 | 0.160 | **0.093** | 0.000 |  |  |
| **D** | 0.333 | 0.436 | 0.243 | 0.382 | **0.441** | 0.384 | 0.000 |  |
| **Y** | 0.301 | 0.401 | 0.196 | 0.342 | 0.387 | 0.334 | 0.259 | 0.000 |

**Table S4.** Block Statistics for HB Breed

| SSC | Blocks  (*n*) | Total block length (kb) | Block size(*kb*) | | | No. of SNPs in blocks(*n*) | SNPs (*n*) | | | % of SNPs in blocks |
| --- | --- | --- | --- | --- | --- | --- | --- | --- | --- | --- |
|  |  |  | mean | min | max |  | mean | min | max |  |
| 1 | 2248 | 30192.27 | 13.43 | 0.002 | 199.97 | 9286 | 4.13 | 2 | 24 | 7.90 |
| 2 | 1710 | 21614.43 | 12.64 | 0.002 | 199.14 | 7603 | 4.45 | 2 | 48 | 6.47 |
| 3 | 1968 | 23015.21 | 11.70 | 0.002 | 198.58 | 9038 | 4.59 | 2 | 29 | 7.69 |
| 4 | 1419 | 18822.96 | 13.27 | 0.002 | 198.84 | 6374 | 4.49 | 2 | 29 | 5.42 |
| 5 | 1344 | 10963.91 | 8.16 | 0.002 | 197.75 | 5629 | 4.19 | 2 | 28 | 4.79 |
| 6 | 2604 | 36218.36 | 13.91 | 0.002 | 199.91 | 12403 | 4.76 | 2 | 40 | 10.55 |
| 7 | 1672 | 12969.78 | 7.76 | 0.002 | 199.61 | 6958 | 4.16 | 2 | 33 | 5.92 |
| 8 | 1186 | 11734.14 | 9.89 | 0.002 | 196.04 | 4825 | 4.07 | 2 | 17 | 4.10 |
| 9 | 1618 | 15089.09 | 9.32 | 0.002 | 195.61 | 7020 | 4.34 | 2 | 30 | 5.97 |
| 10 | 1214 | 7481.79 | 6.16 | 0.002 | 199.13 | 5090 | 4.19 | 2 | 33 | 4.33 |
| 11 | 753 | 6150.67 | 8.17 | 0.002 | 198.11 | 3083 | 4.09 | 2 | 33 | 2.62 |
| 12 | 1308 | 8738.08 | 6.68 | 0.002 | 186.38 | 5797 | 4.43 | 2 | 28 | 4.93 |
| 13 | 1497 | 22855.45 | 15.27 | 0.002 | 199.63 | 6503 | 4.34 | 2 | 22 | 5.53 |
| 14 | 2220 | 27975.01 | 12.6 | 0.002 | 199.95 | 10056 | 4.53 | 2 | 34 | 8.55 |
| 15 | 1128 | 17296.14 | 15.33 | 0.002 | 199.70 | 4794 | 4.25 | 2 | 27 | 4.08 |
| 16 | 890 | 7620.03 | 8.56 | 0.002 | 195.55 | 3624 | 4.07 | 2 | 29 | 3.08 |
| 17 | 879 | 12894.36 | 14.67 | 0.002 | 197.69 | 4230 | 4.81 | 2 | 32 | 3.60 |
| 18 | 961 | 9198.03 | 9.57 | 0.002 | 198.63 | 4064 | 4.23 | 2 | 25 | 3.46 |
| X | 281 | 6487.41 | 23.09 | 0.002 | 198.11 | 1172 | 4.17 | 2 | 21 | 1.00 |
| **Total** | **26900** | **307317.12** | **11.59** |  |  | **117549** |  |  |  | **100.00** |

**Table S5.** Block Statistics for SZ Breed

| SSC | Blocks  (*n*) | Total block length (kb) | Block size(*kb*) | | | No. of SNPs in blocks(*n*) | SNPs (*n*) | | | % of SNPs in blocks |
| --- | --- | --- | --- | --- | --- | --- | --- | --- | --- | --- |
|  |  |  | mean | min | max |  | mean | min | max |  |
| 1 | 1609 | 13468.34 | 8.37 | 0.002 | 199.86 | 6289 | 3.91 | 2 | 26 | 8.33 |
| 2 | 1156 | 5353.14 | 4.63 | 0.002 | 189.24 | 4351 | 3.76 | 2 | 23 | 5.76 |
| 3 | 1561 | 8860.28 | 5.68 | 0.002 | 199.81 | 6120 | 3.92 | 2 | 22 | 8.11 |
| 4 | 1115 | 4980.46 | 4.47 | 0.002 | 184.80 | 4275 | 3.83 | 2 | 20 | 5.66 |
| 5 | 1010 | 4934.01 | 4.89 | 0.002 | 197.38 | 3932 | 3.89 | 2 | 22 | 5.21 |
| 6 | 1751 | 10351.67 | 5.91 | 0.002 | 197.06 | 6966 | 3.98 | 2 | 39 | 9.23 |
| 7 | 1180 | 3372.95 | 2.86 | 0.002 | 174.22 | 4391 | 3.72 | 2 | 24 | 5.82 |
| 8 | 850 | 3450.22 | 4.06 | 0.002 | 196.68 | 3178 | 3.74 | 2 | 21 | 4.21 |
| 9 | 1200 | 4856.43 | 4.05 | 0.002 | 194.52 | 4767 | 3.97 | 2 | 27 | 6.31 |
| 10 | 851 | 2836.97 | 3.33 | 0.002 | 182.79 | 3207 | 3.77 | 2 | 18 | 4.25 |
| 11 | 633 | 1603.38 | 2.53 | 0.002 | 148.71 | 2304 | 3.64 | 2 | 20 | 3.05 |
| 12 | 926 | 2417.31 | 2.61 | 0.002 | 137.13 | 3584 | 3.87 | 2 | 20 | 4.75 |
| 13 | 1061 | 4822.82 | 4.55 | 0.002 | 195.05 | 4059 | 3.83 | 2 | 20 | 5.38 |
| 14 | 1490 | 7509.94 | 5.04 | 0.002 | 197.68 | 6045 | 4.06 | 2 | 38 | 8.01 |
| 15 | 938 | 6759.04 | 7.2 | 0.002 | 197.44 | 3758 | 4.01 | 2 | 34 | 4.98 |
| 16 | 656 | 2302.24 | 3.51 | 0.002 | 198.87 | 2276 | 3.47 | 2 | 17 | 3.01 |
| 17 | 763 | 2275.68 | 2.98 | 0.002 | 105.57 | 2778 | 3.64 | 2 | 19 | 3.68 |
| 18 | 612 | 2044.03 | 3.34 | 0.002 | 180.12 | 2349 | 3.84 | 2 | 19 | 3.11 |
| X | 223 | 2745.46 | 12.31 | 0.002 | 180.14 | 871 | 3.91 | 2 | 20 | 1.15 |
| **Total** | **19585** | **94944.37** | **4.86** |  |  | **75500** |  |  |  | **100.00** |

**Table S6**. Block Statistics for HD Breed

| SSC | Blocks  (*n*) | Total block length (kb) | Block size(*kb*) | | | No. of SNPs in blocks(*n*) | SNPs (*n*) | | | % of SNPs in blocks |
| --- | --- | --- | --- | --- | --- | --- | --- | --- | --- | --- |
|  |  |  | mean | min | max |  | mean | min | max |  |
| 1 | 2131 | 35576.20 | 16.70 | 0.002 | 199.92 | 9159 | 4.3 | 2 | 33 | 7.98 |
| 2 | 1584 | 19322.14 | 12.20 | 0.002 | 199.36 | 6842 | 4.32 | 2 | 36 | 5.96 |
| 3 | 1900 | 21080.08 | 11.10 | 0.002 | 199.73 | 8663 | 4.56 | 2 | 39 | 7.55 |
| 4 | 1387 | 25729.14 | 18.55 | 0.002 | 199.77 | 6764 | 4.88 | 2 | 39 | 5.89 |
| 5 | 1395 | 11739.55 | 8.42 | 0.002 | 199.27 | 5596 | 4.01 | 2 | 23 | 4.87 |
| 6 | 2535 | 38282.92 | 15.10 | 0.002 | 199.79 | 11738 | 4.63 | 2 | 38 | 10.23 |
| 7 | 1594 | 21933.12 | 13.76 | 0.002 | 198.74 | 7280 | 4.57 | 2 | 35 | 6.34 |
| 8 | 1061 | 20851.84 | 19.65 | 0.002 | 199.96 | 4807 | 4.53 | 2 | 29 | 4.19 |
| 9 | 1596 | 16429.31 | 10.29 | 0.002 | 198.76 | 6868 | 4.3 | 2 | 47 | 5.98 |
| 10 | 1129 | 11467.26 | 10.16 | 0.002 | 198.03 | 5081 | 4.5 | 2 | 33 | 4.43 |
| 11 | 704 | 7016.02 | 9.97 | 0.002 | 192.55 | 3013 | 4.28 | 2 | 27 | 2.62 |
| 12 | 1103 | 13156.03 | 11.93 | 0.002 | 199.03 | 5380 | 4.88 | 2 | 29 | 4.69 |
| 13 | 1378 | 20089.39 | 14.58 | 0.002 | 195.92 | 5898 | 4.28 | 2 | 30 | 5.14 |
| 14 | 1893 | 18017.73 | 9.52 | 0.002 | 199.85 | 8097 | 4.28 | 2 | 28 | 7.05 |
| 15 | 1419 | 17550.12 | 12.37 | 0.002 | 198.69 | 5944 | 4.19 | 2 | 30 | 5.18 |
| 16 | 928 | 8169.06 | 8.80 | 0.002 | 197.56 | 3830 | 4.13 | 2 | 25 | 3.34 |
| 17 | 1145 | 10193.80 | 8.90 | 0.002 | 193.74 | 4814 | 4.2 | 2 | 24 | 4.19 |
| 18 | 763 | 13805.66 | 18.09 | 0.002 | 199.83 | 3988 | 5.23 | 2 | 40 | 3.47 |
| X | 261 | 5610.87 | 21.50 | 0.002 | 199.32 | 1030 | 3.95 | 2 | 22 | 0.90 |
| **Total** | **25906** | **336020.24** | **13.24** |  |  | **114792** |  |  |  | **100.00** |

**Table S7**. Block Statistics for PD Breed

| SSC | Blocks  (*n*) | Total block length (kb) | Block size(*kb*) | | | No. of SNPs in blocks(*n*) | SNPs (*n*) | | | % of SNPs in blocks |
| --- | --- | --- | --- | --- | --- | --- | --- | --- | --- | --- |
|  |  |  | mean | min | max |  | mean | min | max |  |
| 1 | 1294 | 24038.12 | 18.58 | 0.002 | 199.84 | 6407 | 4.95 | 2 | 26 | 8.18 |
| 2 | 992 | 15206.30 | 15.33 | 0.002 | 195.73 | 5131 | 5.17 | 2 | 29 | 6.55 |
| 3 | 1181 | 13413.53 | 11.36 | 0.002 | 194.02 | 6028 | 5.1 | 2 | 33 | 7.70 |
| 4 | 759 | 10808.32 | 14.24 | 0.002 | 198.87 | 3737 | 4.92 | 2 | 32 | 4.77 |
| 5 | 738 | 10018.80 | 13.58 | 0.002 | 198.85 | 4009 | 5.43 | 2 | 28 | 5.12 |
| 6 | 1431 | 21651.95 | 15.13 | 0.002 | 199.74 | 7940 | 5.55 | 2 | 59 | 10.14 |
| 7 | 906 | 10859.24 | 11.99 | 0.002 | 198.61 | 4490 | 4.96 | 2 | 40 | 5.73 |
| 8 | 646 | 12699.02 | 19.66 | 0.002 | 197.16 | 3501 | 5.42 | 2 | 40 | 4.47 |
| 9 | 937 | 11867.72 | 12.66 | 0.002 | 198.64 | 4736 | 5.05 | 2 | 65 | 6.05 |
| 10 | 519 | 5680.05 | 10.94 | 0.002 | 188.65 | 2728 | 5.26 | 2 | 27 | 3.48 |
| 11 | 457 | 6830.72 | 14.95 | 0.002 | 191.68 | 2505 | 5.48 | 2 | 28 | 3.20 |
| 12 | 784 | 6099.48 | 7.78 | 0.002 | 186.41 | 4008 | 5.11 | 2 | 29 | 5.12 |
| 13 | 765 | 11025.88 | 14.41 | 0.002 | 192.99 | 3736 | 4.88 | 2 | 26 | 4.77 |
| 14 | 1171 | 20167.32 | 17.22 | 0.002 | 199.17 | 6584 | 5.62 | 2 | 40 | 8.41 |
| 15 | 745 | 11737.23 | 15.76 | 0.002 | 199.60 | 3975 | 5.34 | 2 | 36 | 5.08 |
| 16 | 461 | 5564.59 | 12.07 | 0.002 | 193.37 | 2488 | 5.4 | 2 | 32 | 3.18 |
| 17 | 616 | 6835.80 | 11.10 | 0.002 | 158.87 | 3035 | 4.93 | 2 | 26 | 3.87 |
| 18 | 459 | 6877.08 | 14.98 | 0.002 | 196.55 | 2691 | 5.86 | 2 | 40 | 3.44 |
| X | 135 | 1959.82 | 14.52 | 0.002 | 162.61 | 594 | 4.4 | 2 | 17 | 0.76 |
| **Total** | **14996** | **213340.97** | **14.01** |  |  | **78323** |  |  |  | **100.00** |

**Table** **S8**. Block Statistics for MMS Breed

| SSC | Blocks  (*n*) | Total block length (kb) | Block size(*kb*) | | | No. of SNPs in blocks(*n*) | SNPs (*n*) | | | % of SNPs in blocks |
| --- | --- | --- | --- | --- | --- | --- | --- | --- | --- | --- |
|  |  |  | mean | min | max |  | mean | min | max |  |
| 1 | 1017 | 20197.80 | 19.86 | 0.002 | 199.84 | 5159 | 5.07 | 2 | 30 | 8.32 |
| 2 | 787 | 10488.37 | 13.33 | 0.002 | 199.56 | 3758 | 4.78 | 2 | 30 | 6.06 |
| 3 | 913 | 11727.92 | 12.85 | 0.002 | 199.97 | 4693 | 5.14 | 2 | 28 | 7.56 |
| 4 | 715 | 9407.75 | 13.16 | 0.002 | 199.38 | 3406 | 4.76 | 2 | 39 | 5.49 |
| 5 | 583 | 8028.20 | 13.77 | 0.002 | 198.25 | 2996 | 5.14 | 2 | 34 | 4.83 |
| 6 | 1236 | 15305.01 | 12.38 | 0.002 | 198.38 | 6274 | 5.08 | 2 | 35 | 10.11 |
| 7 | 816 | 10709.74 | 13.13 | 0.002 | 199.24 | 4020 | 4.93 | 2 | 36 | 6.48 |
| 8 | 491 | 7878.62 | 16.05 | 0.002 | 198.63 | 2267 | 4.62 | 2 | 19 | 3.65 |
| 9 | 831 | 9187.50 | 11.06 | 0.002 | 198.32 | 4117 | 4.95 | 2 | 27 | 6.64 |
| 10 | 487 | 4681.03 | 9.61 | 0.002 | 166.69 | 2603 | 5.34 | 2 | 35 | 4.20 |
| 11 | 369 | 3055.50 | 8.28 | 0.002 | 141.62 | 1683 | 4.56 | 2 | 29 | 2.71 |
| 12 | 545 | 3649.47 | 6.70 | 0.002 | 186.39 | 2638 | 4.84 | 2 | 32 | 4.25 |
| 13 | 614 | 10340.12 | 16.84 | 0.002 | 196.16 | 2953 | 4.81 | 2 | 25 | 4.76 |
| 14 | 1024 | 18008.61 | 17.59 | 0.002 | 197.68 | 5488 | 5.36 | 2 | 35 | 8.85 |
| 15 | 630 | 11647.35 | 18.49 | 0.002 | 199.74 | 3227 | 5.12 | 2 | 37 | 5.20 |
| 16 | 343 | 3584.58 | 10.45 | 0.002 | 199.33 | 1580 | 4.61 | 2 | 17 | 2.55 |
| 17 | 509 | 5131.07 | 10.08 | 0.002 | 198.19 | 2538 | 4.99 | 2 | 54 | 4.09 |
| 18 | 402 | 4850.37 | 12.07 | 0.002 | 199.59 | 2089 | 5.2 | 2 | 23 | 3.37 |
| X | 115 | 2616.10 | 22.75 | 0.002 | 179.36 | 552 | 4.8 | 2 | 23 | 0.89 |
| **Total** | **12427** | **170495.11** | **13.60** |  |  | **62041** |  |  |  | **100.00** |

**Table S9**. Block Statistics for Duroc Breed

| SSC | Blocks  (*n*) | Total block length (kb) | Block size(*kb*) | | | No. of SNPs in blocks(*n*) | SNPs (*n*) | | | % of SNPs in blocks |
| --- | --- | --- | --- | --- | --- | --- | --- | --- | --- | --- |
|  |  |  | mean | min | max |  | mean | min | max |  |
| 1 | 198 | 3349.17 | 16.92 | 0.004 | 199.11 | 1183 | 5.97 | 3 | 86 | 9.63 |
| 2 | 175 | 1968.90 | 11.25 | 0.003 | 157.10 | 953 | 5.45 | 3 | 56 | 7.76 |
| 3 | 183 | 5213.22 | 28.49 | 0.003 | 199.97 | 1331 | 7.27 | 3 | 48 | 10.84 |
| 4 | 108 | 2615.96 | 24.22 | 0.003 | 199.54 | 513 | 4.75 | 3 | 26 | 4.18 |
| 5 | 102 | 1294.48 | 12.69 | 0.003 | 196.90 | 527 | 5.17 | 3 | 34 | 4.29 |
| 6 | 183 | 2365.73 | 12.93 | 0.003 | 186.19 | 1078 | 5.89 | 3 | 58 | 8.78 |
| 7 | 159 | 1740.48 | 10.95 | 0.006 | 178.41 | 778 | 4.89 | 3 | 39 | 6.33 |
| 8 | 99 | 1400.48 | 14.15 | 0.005 | 182.43 | 449 | 4.54 | 3 | 18 | 3.66 |
| 9 | 143 | 1324.12 | 9.26 | 0.005 | 141.46 | 685 | 4.79 | 3 | 28 | 5.58 |
| 10 | 99 | 625.56 | 6.32 | 0.006 | 192.64 | 451 | 4.56 | 3 | 31 | 3.67 |
| 11 | 83 | 1147.07 | 13.83 | 0.004 | 171.84 | 389 | 4.69 | 3 | 28 | 3.17 |
| 12 | 110 | 777.64 | 7.07 | 0.005 | 167.27 | 510 | 4.64 | 3 | 18 | 4.15 |
| 13 | 147 | 3945.25 | 26.84 | 0.007 | 199.27 | 801 | 5.45 | 3 | 31 | 6.52 |
| 14 | 141 | 805.01 | 5.71 | 0.005 | 185.36 | 663 | 4.7 | 3 | 34 | 5.40 |
| 15 | 110 | 1223.17 | 11.12 | 0.006 | 171.60 | 458 | 4.16 | 3 | 14 | 3.73 |
| 16 | 83 | 1314.57 | 15.84 | 0.005 | 198.60 | 477 | 5.75 | 3 | 48 | 3.88 |
| 17 | 96 | 1072.12 | 11.17 | 0.004 | 191.98 | 496 | 5.17 | 3 | 24 | 4.04 |
| 18 | 67 | 858.61 | 12.82 | 0.004 | 180.92 | 338 | 5.04 | 3 | 22 | 2.75 |
| X | 32 | 1505.72 | 47.05 | 0.039 | 182.37 | 203 | 6.34 | 3 | 18 | 1.65 |
| **Total** | **2318** | **34547.26** | **15.72** |  |  | **12283** |  |  |  | **100.00** |

**Table S10**. Block Statistics for Yorkshire Breed

| SSC | Blocks  (*n*) | Total block length (kb) | Block size(*kb*) | | | No. of SNPs in blocks(*n*) | SNPs (*n*) | | | % of SNPs in blocks |
| --- | --- | --- | --- | --- | --- | --- | --- | --- | --- | --- |
|  |  |  | mean | min | max |  | mean | min | max |  |
| 1 | 1300 | 15888.23 | 12.22 | 0.002 | 198.71 | 5807 | 4.47 | 2 | 46 | 7.56 |
| 2 | 1019 | 11418.51 | 11.21 | 0.002 | 197.72 | 4503 | 4.42 | 2 | 29 | 5.86 |
| 3 | 1207 | 10396.05 | 8.61 | 0.002 | 197.03 | 5483 | 4.54 | 2 | 26 | 7.13 |
| 4 | 907 | 12682.92 | 13.98 | 0.002 | 199.50 | 4386 | 4.84 | 2 | 65 | 5.71 |
| 5 | 839 | 8247.50 | 9.83 | 0.002 | 199.96 | 3920 | 4.67 | 2 | 46 | 5.10 |
| 6 | 1702 | 16343.17 | 9.60 | 0.002 | 199.85 | 7799 | 4.58 | 2 | 35 | 10.15 |
| 7 | 947 | 6464.60 | 6.83 | 0.002 | 198.42 | 4140 | 4.37 | 2 | 32 | 5.39 |
| 8 | 842 | 8416.56 | 10.00 | 0.002 | 199.12 | 3465 | 4.12 | 2 | 24 | 4.51 |
| 9 | 1012 | 10819.64 | 10.69 | 0.002 | 199.32 | 4629 | 4.57 | 2 | 27 | 6.02 |
| 10 | 715 | 4972.55 | 6.96 | 0.002 | 182.79 | 3272 | 4.58 | 2 | 37 | 4.26 |
| 11 | 588 | 4940.91 | 8.40 | 0.002 | 196.66 | 2515 | 4.28 | 2 | 27 | 3.27 |
| 12 | 894 | 7685.64 | 8.60 | 0.002 | 199.27 | 4421 | 4.95 | 2 | 48 | 5.75 |
| 13 | 916 | 17384.64 | 18.98 | 0.002 | 199.58 | 4282 | 4.67 | 2 | 36 | 5.57 |
| 14 | 1266 | 14087.01 | 11.13 | 0.002 | 199.91 | 6107 | 4.82 | 2 | 54 | 7.95 |
| 15 | 771 | 9996.37 | 12.97 | 0.002 | 199.26 | 3481 | 4.51 | 2 | 31 | 4.53 |
| 16 | 456 | 4898.86 | 10.74 | 0.002 | 194.75 | 2070 | 4.54 | 2 | 33 | 2.69 |
| 17 | 770 | 5527.96 | 7.18 | 0.002 | 197.37 | 3344 | 4.34 | 2 | 27 | 4.35 |
| 18 | 498 | 6174.18 | 12.4 | 0.002 | 196.40 | 2373 | 4.77 | 2 | 28 | 3.09 |
| X | 216 | 3403.50 | 15.76 | 0.002 | 199.44 | 859 | 3.98 | 2 | 17 | 1.12 |
| **Total** | **16865** | **179748.80** | **10.85** |  |  | **76856** |  |  |  | **100.00** |

**Table S11.** The percentage of genome covered by blocks in each breed.

| **SSC** | **BREEDS** | | | | | | | |
| --- | --- | --- | --- | --- | --- | --- | --- | --- |
|  | **JQ** | **HB** | **HD** | **SZ** | **MMS** | **PD** | **D** | **Y** |
| 1 | 15.74 | 11.01 | 12.97 | 4.91 | 7.36 | 8.76 | 1.22 | 5.79 |
| 2 | 18.93 | 14.23 | 12.72 | 3.52 | 6.90 | 10.01 | 1.30 | 7.52 |
| 3 | 18.70 | 17.32 | 15.87 | 6.67 | 8.83 | 10.10 | 3.92 | 7.83 |
| 4 | 13.87 | 14.38 | 19.65 | 3.80 | 7.19 | 8.26 | 2.00 | 9.69 |
| 5 | 13.77 | 10.49 | 11.23 | 4.72 | 7.68 | 9.58 | 1.24 | 7.89 |
| 6 | 20.14 | 21.20 | 22.41 | 6.06 | 8.96 | 12.67 | 1.38 | 9.57 |
| 7 | 14.17 | 10.64 | 18.00 | 2.77 | 8.79 | 8.91 | 1.43 | 5.31 |
| 8 | 9.68 | 8.44 | 15.00 | 2.48 | 5.67 | 9.14 | 1.01 | 6.06 |
| 9 | 16.31 | 10.82 | 11.78 | 3.48 | 6.59 | 8.51 | 0.95 | 7.76 |
| 10 | 12.77 | 10.79 | 16.53 | 4.09 | 6.75 | 8.19 | 0.90 | 7.17 |
| 11 | 12.60 | 7.77 | 8.86 | 2.03 | 3.86 | 8.63 | 1.45 | 6.24 |
| 12 | 13.58 | 14.19 | 21.36 | 3.92 | 5.92 | 9.90 | 1.26 | 12.48 |
| 13 | 10.78 | 10.97 | 9.64 | 2.31 | 4.96 | 5.29 | 1.89 | 8.34 |
| 14 | 18.03 | 19.73 | 12.71 | 5.30 | 12.70 | 14.23 | 0.57 | 9.94 |
| 15 | 17.15 | 12.32 | 12.50 | 4.81 | 8.30 | 8.36 | 0.87 | 7.12 |
| 16 | 13.93 | 9.53 | 10.22 | 2.88 | 4.48 | 6.96 | 1.64 | 6.13 |
| 17 | 15.89 | 20.31 | 16.06 | 3.58 | 8.08 | 10.77 | 1.69 | 8.71 |
| 18 | 13.51 | 16.43 | 24.66 | 3.65 | 8.66 | 12.28 | 1.53 | 11.03 |
| X | 3.52 | 5.15 | 4.46 | 2.18 | 2.08 | 1.56 | 1.20 | 2.70 |
| **Total (%)** | **14.36** | **12.62** | **13.80** | **3.90** | **7.00** | **8.76** | **1.42** | **7.38** |

**NB:** Maximum genome covered across breed is colored in blue.

**Table S12.** List of the 88 overlapping genes detected in the JQ blocks.

| **SSC** | **Start position** | **End position** | **Width(bp)** | **No. of Genes** | **Gene ID** | | **Gene Symbol** |
| --- | --- | --- | --- | --- | --- | --- | --- |
| 1 | 57754743 | 57954471 | 199729 | 4 | ENSSSCG00000034904 | |  |
|  |  |  |  |  | ENSSSCG00000035516 | |  |
|  |  |  |  |  | ENSSSCG00000004331 | | CASP8AP2 |
|  |  |  |  |  | ENSSSCG00000004330 | | GJA10 |
| 1 | 145630575 | 145788505 | 157931 | 1 | ENSSSCG00000005786 | | SNRPA1 |
| 2 | 150363020 | 150561606 | 198587 | 4 | ENSSSCG00000014431 | | AFAP1L1 |
|  |  |  |  |  | ENSSSCG00000014432 | | GRPEL2 |
|  |  |  |  |  | ENSSSCG00000014433 | | PCYOX1L |
|  |  |  |  |  | ENSSSCG00000014434 | | IL17B |
| 5 | 17274912 | 17439428 | 164517 | 6 | ENSSSCG00000000232 | | ACVRL1 |
|  |  |  |  |  | ENSSSCG00000000233 | | ACVR1B |
|  |  |  |  |  | ENSSSCG00000000234 | | GRASP |
|  |  |  |  |  | ENSSSCG00000031321 | | NR4A1 |
|  |  |  |  |  | ENSSSCG00000033687 | | ATG101 |
|  |  |  |  |  | ENSSSCG00000035962 |  | |
| 6 | 48308117 | 48507976 | 199860 | 6 | ENSSSCG00000030510 | | FBL |
|  |  |  |  |  | ENSSSCG00000027515 | | FCGBP |
|  |  |  |  |  | ENSSSCG00000033837 |  | |
|  |  |  |  |  | ENSSSCG00000039235 | | PSMC4 |
|  |  |  |  |  | ENSSSCG00000002988 |  | |
|  |  |  |  |  | ENSSSCG00000018799 | | U6 |
| 6 | 52898408 | 53098319 | 199912 | 4 | ENSSSCG00000003111 | | SAE1 |
|  |  |  |  |  | ENSSSCG00000003112 |  | |
|  |  |  |  |  | ENSSSCG00000024240 | | CCDC9 |
|  |  |  |  |  | ENSSSCG00000037986 | | INAFM1 |
| 6 | 64203148 | 64347705 | 144558 | 3 | ENSSSCG00000028706 | | MORN1 |
|  |  |  |  |  | ENSSSCG00000026626 | | RER1 |
|  |  |  |  |  | ENSSSCG00000027916 | | PEX10 |
| 6 | 65204715 | 65346283 | 141569 | 9 | ENSSSCG00000040858 |  | |
|  |  |  |  |  | ENSSSCG00000034255 |  | |
|  |  |  |  |  | ENSSSCG00000039233 |  | |
|  |  |  |  |  | ENSSSCG00000036305 |  | |
|  |  |  |  |  | ENSSSCG00000039516 | | LRRC47 |
|  |  |  |  |  | ENSSSCG00000003362 | | CEP104 |
|  |  |  |  |  | ENSSSCG00000020449 | | ssc-mir-2320 |
|  |  |  |  |  | ENSSSCG00000021436 | | DFFB |
|  |  |  |  |  | ENSSSCG00000031102 | | C1orf174 |
| 7 | 57703699 | 57797277 | 93579 | 1 | ENSSSCG00000001872 | | LINGO1 |
| 13 | 71557016 | 71756980 | 199965 | 6 | ENSSSCG00000031176 | | GP9 |
|  |  |  |  |  | ENSSSCG00000037568 | | RAB43 |
|  |  |  |  |  | ENSSSCG00000038900 |  | |
|  |  |  |  |  | ENSSSCG00000040879 | | CNBP |
|  |  |  |  |  | ENSSSCG00000011616 | | COPG1 |
|  |  |  |  |  | ENSSSCG00000011611 | | HMCES |
| 14 | 47714888 | 47914794 | 199907 | 8 | ENSSSCG00000010016 | | MORC2 |
|  |  |  |  |  | ENSSSCG00000036935 | | TUG1_1 |
|  |  |  |  |  | ENSSSCG00000035042 | | TUG1_2 |
|  |  |  |  |  | ENSSSCG00000040413 | | TUG1_3 |
|  |  |  |  |  | ENSSSCG00000038425 | | TUG1_4 |
|  |  |  |  |  | ENSSSCG00000010017 | | SMTN |
|  |  |  |  |  | ENSSSCG00000040755 |  | |
|  |  |  |  |  | ENSSSCG00000029781 | | SELENOM |
| 14 | 49585363 | 49722310 | 136948 | 5 | ENSSSCG00000010057 | | LRRC75B |
|  |  |  |  |  | ENSSSCG00000010056 |  | |
|  |  |  |  |  | ENSSSCG00000010055 | | GGT5 |
|  |  |  |  |  | ENSSSCG00000025393 | | SUSD2 |
|  |  |  |  |  | ENSSSCG00000028151 | | 5S_rRNA |
| 14 | 50940408 | 51140288 | 199881 | 10 | ENSSSCG00000010107 | | MED15 |
|  |  |  |  |  | ENSSSCG00000010113 | | TSSK2 |
|  |  |  |  |  | ENSSSCG00000010114 | | ESS2 |
|  |  |  |  |  | ENSSSCG00000010115 | | GSC2 |
|  |  |  |  |  | ENSSSCG00000010116 | | SLC25A1 |
|  |  |  |  |  | ENSSSCG00000010117 |  | |
|  |  |  |  |  | ENSSSCG00000010118 | | HIRA |
|  |  |  |  |  | ENSSSCG00000034014 | | MRPL40 |
|  |  |  |  |  | ENSSSCG00000040740 | | C22orf39 |
|  |  |  |  |  | ENSSSCG00000010121 | | UFD1 |
| 14 | 51322222 | 51522048 | 199827 | 15 | ENSSSCG00000035643 |  | |
|  |  |  |  |  | ENSSSCG00000010131 | | TXNRD2 |
|  |  |  |  |  | ENSSSCG00000010132 | | COMT |
|  |  |  |  |  | ENSSSCG00000010133 | | ARVCF |
|  |  |  |  |  | ENSSSCG00000019684 | | ssc-mir-185 |
|  |  |  |  |  | ENSSSCG00000010135 | | DGCR8 |
|  |  |  |  |  | ENSSSCG00000010134 | | TANGO2 |
|  |  |  |  |  | ENSSSCG00000029742 | | MIR3618 |
|  |  |  |  |  | ENSSSCG00000019500 | | ssc-mir-1306 |
|  |  |  |  |  | ENSSSCG00000010136 | | TRMT2A |
|  |  |  |  |  | ENSSSCG00000039739 |  | |
|  |  |  |  |  | ENSSSCG00000038308 | | RANBP1 |
|  |  |  |  |  | ENSSSCG00000018761 | | SNORA77 |
|  |  |  |  |  | ENSSSCG00000010138 | | ZDHHC8 |
|  |  |  |  |  | ENSSSCG00000010139 | | CCDC188 |
| 17 | 62089019 | 62255688 | 166670 | 5 | ENSSSCG00000038631 | | TCFL5 |
|  |  |  |  |  | ENSSSCG00000033946 | | DIDO1 |
|  |  |  |  |  | ENSSSCG00000034173 | | GID8 |
|  |  |  |  |  | ENSSSCG00000040931 | | SLC17A9 |
|  |  |  |  |  | ENSSSCG00000031937 | | BHLHE23 |
| 18 | 923784 | 1002867 | 79084 | 1 | ENSSSCG00000026661 | | PTPRN2 |

**Table S13.** List of the 80 SNPs in the haplotype block with the maximum number of SNPs in the JQ breed

| **No.** | **Name** | **Position** | **ObsHET** | **PredHET** | **HWpvalue** | **%Genotype** | **MAF** | **Alleles** |
| --- | --- | --- | --- | --- | --- | --- | --- | --- |
| 1 | snp302189 | 923784 | 0.421 | 0.45 | 0.8938 | 100 | 0.342 | C:T |
| 2 | snp302191 | 932798 | 0.211 | 0.188 | 1 | 100 | 0.105 | C:T |
| 3 | snp302192 | 943130 | 0.237 | 0.209 | 1 | 100 | 0.118 | A:G |
| 4 | snp302193 | 943134 | 0.237 | 0.209 | 1 | 100 | 0.118 | A:G |
| 5 | snp302195 | 945293 | 0.237 | 0.209 | 1 | 100 | 0.118 | A:G |
| 6 | snp302196 | 945297 | 0.237 | 0.248 | 1 | 100 | 0.145 | T:C |
| 7 | snp302197 | 945335 | 0.211 | 0.188 | 1 | 100 | 0.105 | T:A |
| 8 | snp302198 | 945511 | 0.395 | 0.472 | 0.4503 | 100 | 0.382 | C:T |
| 9 | snp302199 | 954730 | 0.211 | 0.188 | 1 | 100 | 0.105 | C:T |
| 10 | snp302200 | 984514 | 0.132 | 0.123 | 1 | 100 | 0.066 | C:T |
| 11 | snp302201 | 984602 | 0.263 | 0.229 | 1 | 100 | 0.132 | C:T |
| 12 | snp302202 | 984605 | 0.211 | 0.188 | 1 | 100 | 0.105 | G:T |
| 13 | snp302203 | 984615 | 0.263 | 0.229 | 1 | 100 | 0.132 | G:A |
| 14 | snp302204 | 984623 | 0.263 | 0.229 | 1 | 100 | 0.132 | C:T |
| 15 | snp302205 | 984671 | 0.211 | 0.188 | 1 | 100 | 0.105 | T:C |
| 16 | snp302206 | 984673 | 0.211 | 0.188 | 1 | 100 | 0.105 | G:T |
| 17 | snp302207 | 984710 | 0.211 | 0.188 | 1 | 100 | 0.105 | G:T |
| 18 | snp302208 | 984715 | 0.211 | 0.188 | 1 | 100 | 0.105 | T:A |
| 19 | snp302209 | 984793 | 0.474 | 0.361 | 0.1405 | 100 | 0.237 | A:G |
| 20 | snp302210 | 984848 | 0.211 | 0.188 | 1 | 100 | 0.105 | A:G |
| 21 | snp302211 | 989220 | 0.263 | 0.229 | 1 | 100 | 0.132 | C:T |
| 22 | snp302212 | 989247 | 0.263 | 0.229 | 1 | 100 | 0.132 | G:A |
| 23 | snp302213 | 989250 | 0.184 | 0.167 | 1 | 100 | 0.092 | G:C |
| 24 | snp302214 | 989344 | 0.263 | 0.229 | 1 | 100 | 0.132 | G:A |
| 25 | snp302215 | 989356 | 0.263 | 0.229 | 1 | 100 | 0.132 | A:T |
| 26 | snp302216 | 989359 | 0.263 | 0.229 | 1 | 100 | 0.132 | G:C |
| 27 | snp302217 | 989361 | 0.263 | 0.229 | 1 | 100 | 0.132 | A:G |
| 28 | snp302218 | 989372 | 0.263 | 0.229 | 1 | 100 | 0.132 | T:C |
| 29 | snp302219 | 989373 | 0.263 | 0.229 | 1 | 100 | 0.132 | G:A |
| 30 | snp302220 | 989376 | 0.263 | 0.229 | 1 | 100 | 0.132 | G:C |
| 31 | snp302221 | 991257 | 0.211 | 0.188 | 1 | 100 | 0.105 | C:T |
| 32 | snp302222 | 991333 | 0.5 | 0.5 | 1 | 100 | 0.487 | C:T |
| 33 | snp302223 | 991468 | 0.474 | 0.388 | 0.3876 | 100 | 0.263 | A:G |
| 34 | snp302224 | 992417 | 0.395 | 0.458 | 0.5542 | 100 | 0.355 | G:C |
| 35 | snp302225 | 992511 | 0.263 | 0.229 | 1 | 100 | 0.132 | A:G |
| 36 | snp302226 | 992525 | 0.263 | 0.229 | 1 | 100 | 0.132 | G:T |
| 37 | snp302227 | 992554 | 0.184 | 0.209 | 0.8283 | 100 | 0.118 | G:T |
| 38 | snp302228 | 992557 | 0.184 | 0.209 | 0.8283 | 100 | 0.118 | T:C |
| 39 | snp302229 | 992603 | 0.421 | 0.388 | 1 | 100 | 0.263 | C:T |
| 40 | snp302230 | 992604 | 0.474 | 0.411 | 0.6604 | 100 | 0.289 | G:A |
| 41 | snp302231 | 992612 | 0.474 | 0.411 | 0.6604 | 100 | 0.289 | C:T |
| 42 | snp302232 | 992614 | 0.368 | 0.45 | 0.3944 | 100 | 0.342 | T:G |
| 43 | snp302234 | 992700 | 0.263 | 0.266 | 1 | 100 | 0.158 | G:C |
| 44 | snp302235 | 992714 | 0.395 | 0.441 | 0.7017 | 100 | 0.329 | A:G |
| 45 | snp302236 | 992750 | 0.5 | 0.4 | 0.2838 | 100 | 0.276 | A:G |
| 46 | snp302237 | 992787 | 0.5 | 0.5 | 1 | 100 | 0.487 | G:A |
| 47 | snp302238 | 992801 | 0.395 | 0.441 | 0.7017 | 100 | 0.329 | T:C |
| 48 | snp302239 | 993897 | 0.474 | 0.388 | 0.3876 | 100 | 0.263 | G:A |
| 49 | snp302240 | 994556 | 0.237 | 0.248 | 1 | 100 | 0.145 | G:A |
| 50 | snp302241 | 994564 | 0.237 | 0.248 | 1 | 100 | 0.145 | A:G |
| 51 | snp302242 | 994578 | 0.237 | 0.248 | 1 | 100 | 0.145 | C:T |
| 52 | snp302243 | 994581 | 0.237 | 0.248 | 1 | 100 | 0.145 | T:C |
| 53 | snp302244 | 994595 | 0.368 | 0.45 | 0.3944 | 100 | 0.342 | G:A |
| 54 | snp302245 | 994601 | 0.447 | 0.472 | 0.9468 | 100 | 0.382 | T:C |
| 55 | snp302246 | 994669 | 0.421 | 0.465 | 0.7413 | 100 | 0.368 | C:T |
| 56 | snp302247 | 995018 | 0.5 | 0.491 | 1 | 100 | 0.434 | G:A |
| 57 | snp302248 | 995030 | 0.237 | 0.248 | 1 | 100 | 0.145 | C:T |
| 58 | snp302249 | 995056 | 0.447 | 0.483 | 0.8365 | 100 | 0.408 | G:A |
| 59 | snp302250 | 995079 | 0.237 | 0.248 | 1 | 100 | 0.145 | T:G |
| 60 | snp302252 | 996103 | 0.474 | 0.478 | 1 | 100 | 0.395 | G:A |
| 61 | snp302253 | 999244 | 0.184 | 0.167 | 1 | 100 | 0.092 | C:T |
| 62 | snp302254 | 999406 | 0.447 | 0.472 | 0.9468 | 100 | 0.382 | G:T |
| 63 | snp302255 | 999426 | 0.237 | 0.248 | 1 | 100 | 0.145 | G:A |
| 64 | snp302256 | 999490 | 0.237 | 0.248 | 1 | 100 | 0.145 | T:C |
| 65 | snp302257 | 999542 | 0.237 | 0.248 | 1 | 100 | 0.145 | A:G |
| 66 | snp302258 | 999699 | 0.237 | 0.248 | 1 | 100 | 0.145 | C:G |
| 67 | snp302259 | 999778 | 0.553 | 0.422 | 0.1355 | 100 | 0.303 | T:C |
| 68 | snp302260 | 999811 | 0.237 | 0.248 | 1 | 100 | 0.145 | G:C |
| 69 | snp302261 | 999838 | 0.237 | 0.248 | 1 | 100 | 0.145 | G:A |
| 70 | snp302262 | 1001581 | 0.263 | 0.266 | 1 | 100 | 0.158 | G:A |
| 71 | snp302264 | 1001590 | 0.263 | 0.266 | 1 | 100 | 0.158 | G:A |
| 72 | snp302265 | 1001606 | 0.263 | 0.266 | 1 | 100 | 0.158 | T:C |
| 73 | snp302266 | 1001669 | 0.263 | 0.266 | 1 | 100 | 0.158 | A:C |
| 74 | snp302267 | 1001671 | 0.263 | 0.266 | 1 | 100 | 0.158 | C:T |
| 75 | snp302268 | 1001681 | 0.263 | 0.266 | 1 | 100 | 0.158 | C:T |
| 76 | snp302269 | 1001717 | 0.5 | 0.483 | 1 | 100 | 0.408 | G:A |
| 77 | snp302270 | 1001835 | 0.237 | 0.284 | 0.5477 | 100 | 0.171 | A:G |
| 78 | snp302271 | 1001872 | 0.237 | 0.284 | 0.5477 | 100 | 0.171 | G:A |
| 79 | snp302272 | 1002796 | 0.579 | 0.432 | 0.0878 | 100 | 0.316 | A:G |
| 80 | snp302273 | 1002867 | 0.474 | 0.499 | 0.9477 | 100 | 0.474 | C:T |

**Table S14**. The statistics of common haplotypes shared across populations (in Mb).

|  | **JQ** | **HB** | **SZ** | **HD** | **MMS** | **PD** | **D** | **Y** |
| --- | --- | --- | --- | --- | --- | --- | --- | --- |
| **JQ** | 345.30 | 96.04 | 41.75 | 89.30 | 64.67 | 76.55 | 8.97 | 44.87 |
| **HB** |  | 300.83 | 32.05 | 81.33 | 54.64 | 62.38 | 8.20 | 38.96 |
| **SZ** |  |  | 92.20 | 25.32 | 21.30 | 25.56 | 2.16 | 16.71 |
| **HD** |  |  |  | 330.41 | 54.29 | 60.40 | 7.78 | 39.65 |
| **MMS** |  |  |  |  | 167.88 | 52.25 | 4.10 | 20.92 |
| **PD** |  |  |  |  |  | 211.38 | 5.18 | 27.90 |
| **D** |  |  |  |  |  |  | 33.04 | 5.04 |
| **Y** |  |  |  |  |  |  |  | 176.35 |

**NB:** The corresponding percentage is presented in Table 3 of the main text.
